# Declines in organic matter persistence with increased soil carbon

**DOI:** 10.1038/s41467-026-75185-4

**Published:** 2026-07-07

**Authors:** Guang Zhao, Chao Liang, Zhihua Liu, Nan Cong, Zhoutao Zheng, Edith Bai, Juntao Zhu, Bo Zhao, Yixuan Zhu, Mengke Cai, Xiaoqing Duan, Hui Wang, Jianbei Huang, Yangjian Zhang, Susan Trumbore

**Affiliations:** 1 Naqu Alpine Grassland Ecosystem National Observation and Research Station, Key Laboratory of Ecosystem Network Observation and Modeling, Institute of Geographic Sciences and Natural Resources Research, Chinese Academy of Sciences, Beijing, China; 2 Department of Biogeochemical Processes, Max Planck Institute for Biogeochemistry, Jena, Germany; 3 College of Environmental and Resource Sciences, Zhejiang A&F University, Hangzhou, China; 4 Institute of Applied Ecology, Chinese Academy of Sciences, Shenyang, China; 5 CAS Key Laboratory of Forest Ecology and Silviculture, Institute of Applied Ecology, Chinese Academy of Sciences, Shenyang, China; 6 School of Geographical Sciences, Northeast Normal University, Changchun, China; 7 School of Life Sciences/Hebei Basic Science Center for Biotic Interaction, Institute of Life Science and Green Development, Hebei University, Baoding, Hebei China; 8 Institute of Geographic Sciences and Natural Resources Research, Chinese Academy of Sciences, Beijing, China; 9 College of Resources and Environment, University of Chinese Academy of Sciences, Beijing, China

**Keywords:** Carbon cycle, Biogeography

## Abstract

The persistence of soil organic matter (SOM) is critical for predicting carbon-climate feedbacks, yet how increasing soil organic carbon (SOC) relates to long-term SOM persistence across environmental gradients remains unclear. Soil radiocarbon, SOM physicochemical composition, and microbial carbon use efficiency (CUE) are analyzed along a precipitation-driven gradient integrated with global datasets. Here we show, as SOC content increases, its persistence as reflected by radiocarbon signatures declines at both the transect and global scales. This pattern indicates that higher SOC soils are increasingly dominated by younger, faster-cycling carbon rather than older, stabilized pools. Plant carbon inputs strongly predict SOM persistence and are associated with younger, less persistent SOC. While microbial CUE increases with SOC, higher CUE does not necessarily enhance long-term stabilization. Our findings suggest that SOC content alone provides limited insight into long-term soil carbon persistence and highlight the importance of explicitly representing plant carbon inputs and microbially mediated persistence in Earth system models.

## Abstract (web summary)

Higher soil organic carbon does not necessarily indicate more persistent carbon storage. Across regional and global datasets, carbon rich soils are dominated by younger, faster-cycling organic matter shaped by plant inputs, microbes and minerals.

## Introduction

Soil organic carbon (SOC) constitutes the largest terrestrial carbon reservoir, and its long-term persistence plays an important role in regulating climate–carbon feedbacks^1,2^. SOC stocks reflect the net balance between carbon inputs from plant production and carbon losses, primarily driven by microbial decomposition and respiration^3^. While increasing plant carbon inputs can potentially enhance SOC stocks, the extent to which these inputs contribute to long-term storage is governed by the persistence of soil organic matter (SOM)^3,4^. Beyond carbon inputs, growing evidence suggests that SOM persistence depends less on chemical recalcitrance alone and more on the complementary effects of mineral protection and the stabilization of microbial-derived compounds^5,6^. However, the relative importance of these processes, and how their influence on SOM persistence varies across environmental gradients, remain poorly understood. As a result, SOC storage alone may provide limited information about underlying SOM persistence, constraining our ability to predict soil carbon responses to global change.

Addressing this question requires determining whether increases in SOC stocks correspond to enhanced SOM persistence. Although SOC stocks may increase under conditions of greater carbon inputs or reduced losses, they do not necessarily reflect the turnover dynamics or effective age of soil carbon^3,7^. For example, high carbon inputs can facilitate rapid SOC accrual while also exhibiting fast cycling, potentially limiting long-term persistence^2,4,8^. Consequently, an independent metric is needed to assess SOM persistence beyond carbon stocks alone. Among available indicators, soil radiocarbon (Δ^14^C) provides an integrative constraint on SOM persistence by reflecting the effective transit time of carbon through the soil system^7,9^. Lower Δ^14^C values generally indicate older, more persistent carbon, whereas higher values reflect a greater contribution of recently fixed, rapidly cycling carbon^10^. Because Δ^14^C captures processes operating over years to millennia and is largely independent of carbon stock size, it has been widely applied to quantify SOM persistence across ecosystems and environmental gradients^11^. Accordingly, Δ^14^C offers a robust framework for evaluating how SOM persistence varies along the gradient of SOC accumulation.

SOM persistence reflects the interplay of multiple stabilization pathways rather than SOC content alone. Among several proposed stabilization pathways, mineral protection through association with soil minerals, particularly reactive Fe/Al oxides, has been regarded as a dominant mechanism for long-term carbon retention by constraining microbial access^12,13^, complemented by the molecular recalcitrance of organic compounds^14,15^. Nevertheless, accumulating evidence indicates that neither mineral association nor molecular-level recalcitrance alone is sufficient to predict SOM persistence across environmental gradients^4,16^. In this context, SOM persistence is increasingly viewed as emerging from a dynamic balance between passive mineral–organic interactions and active microbial regulation^17^. Accordingly, growing attention has focused on the role of microorganisms in regulating SOM persistence, as they transform plant-derived inputs into microbial products and can also facilitate the turnover of previously stabilized carbon pools^5,6,18^. Microbial traits, including community composition, extracellular enzyme allocation, and carbon use efficiency (CUE), mediate the partitioning of organic inputs between particulate and mineral-associated pools and influence whether microbial-derived SOM is retained or rapidly reutilized^19,20^. Specifically, variations in plant carbon inputs and stoichiometric imbalances are increasingly recognized as primary drivers modulating the microbial regulation of SOM persistence^21,22^. Under high-input conditions, intensified microbial demand and activity may shift SOM persistence from mineral protection toward accelerated microbial turnover as SOC accumulates^8,23,24^. Despite this, few studies have evaluated mineral protection, molecular composition, and microbial traits within an integrated framework to assess how their relative contributions to SOM persistence vary along SOC gradients.

**Fig. 1:**
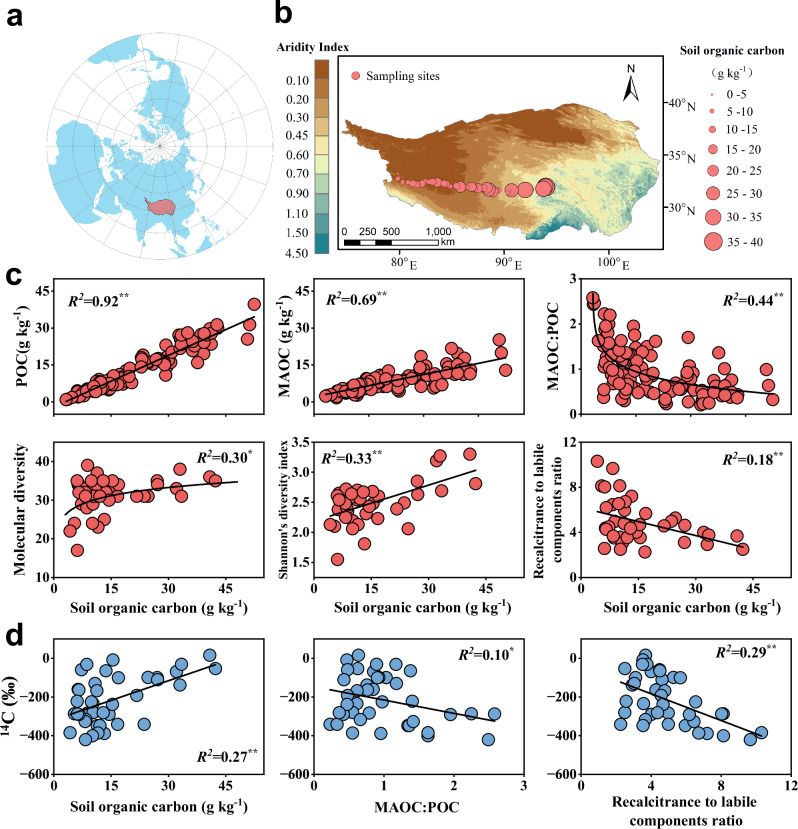
Changes in soil organic matter (SOM) persistence along the transect. **a**, Geographic location of the study area on the Tibetan Plateau. **b**, Distribution of sampling sites along the transect. **c**, Relationships between soil organic carbon (SOC) content and physicochemical properties. **d**, Relationship between soil radiocarbon (Δ^14^C) and soil physicochemical properties. MAOC, mineral-associated organic carbon; POC, particulate organic carbon. MAOC:POC, ratio of mineral-associated carbon to particulate organic carbon. Molecular diversity and Shannon–Wiener indices describe SOM chemical composition. Recalcitrant: labile ratio, ratio of recalcitrant to labile SOM components (see Methods). *P < 0.05; **P < 0.01.

To fill this gap, we evaluate how SOM persistence, as constrained by Δ^14^C signatures, varies with increasing SOC across a precipitation-driven gradient on the Tibetan Plateau (Fig. 1a, b), and simultaneously assess the relative roles of physical, chemical, and microbial stabilization pathways. In this study, Δ^14^C is used as an integrative indicator of SOM persistence, while mineral protection, molecular composition, and microbial traits are evaluated as mechanistic pathways regulating persistence. We hypothesize that SOM persistence declines with increasing SOC content across soil carbon gradients, reflecting the increasing influence of plant carbon inputs and associated microbially mediated soil carbon turnover relative to mineral-associated stabilization. To test the global robustness of these patterns, we integrate our regional data with two comprehensive datasets: the Global Soil Carbon Fractionation Database and the International Soil Radiocarbon Database. Our results reveal a consistent global decline in SOM persistence with increasing SOC, a trend intricately regulated by the interplay among plant carbon inputs, microbial metabolic traits, and mineral protection.

## Results

### SOM composition and microbial properties along the climatic transect over the Tibetan Plateau

The high-altitude grassland transects lie along an east-west gradient that varies more in precipitation (mean annual precipitation: 54–654 mm) than in temperature (mean annual temperature: −3.6 to 2.3 °C; Fig. S1). SOC content (4–40 g kg^−1^) increased as conditions became wetter (i.e., higher aridity index; aridity index, the ratio of annual precipitation to potential evapotranspiration; higher aridity index values indicate wetter conditions; Fig. 1b; Fig. S1). Plant biomass, net primary productivity (NPP), soil total nitrogen (N), pH, dissolved organic carbon and available N (AN) also increased with the aridity index (Table S1). To investigate the controls on soil carbon persistence, we employed a multi-faceted approach that integrated direct measurements of SOM persistence with mechanistic evaluations of three key stabilization pathways. First, we quantified SOM persistence using radiocarbon (Δ^14^C) as a time-integrated indicator of carbon turnover. Then, to understand the potential mechanisms behind the observed persistence patterns, we systematically evaluated:(1) physical protection through density fractionation into particulate and mineral-associated organic matter (POM vs. MAOM);(2) mineral protection by quantifying reactive Fe/Al oxides; and (3) molecular-level recalcitrance via pyrolysis-GC/MS analysis of bulk SOM composition.

**Fig. 2:**
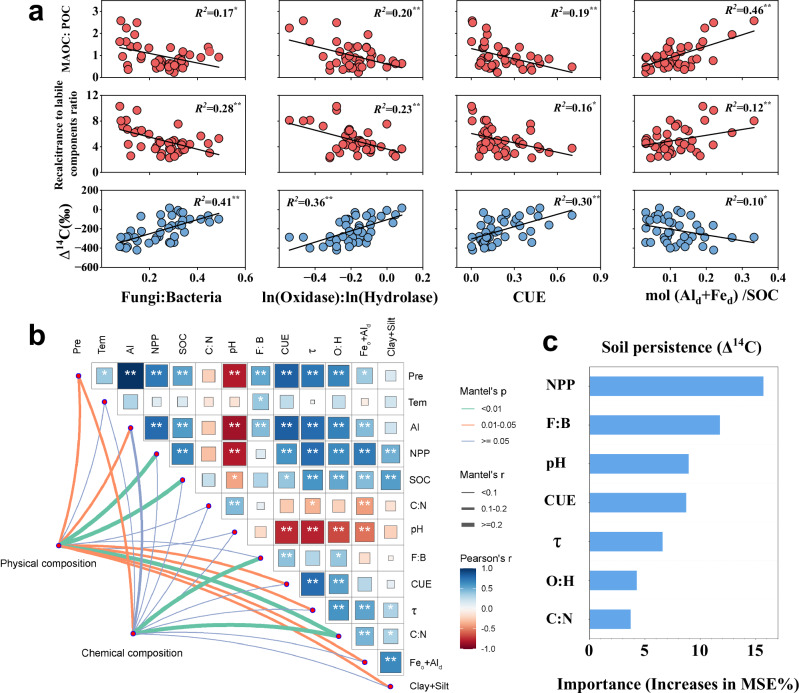
Environmental controls on soil organic matter (SOM) persistence. **a**, Correlations among SOM composition, persistence, microbial properties, and iron-aluminum oxides. **b**, Relationships between SOM physical and chemical composition and environmental drivers. **c**, Relative importance of biotic and abiotic variables influencing SOM persistence revealed by the random forest model. Predictor importance was estimated as the increase in mean square error (MSE); higher values indicate greater importance. MAOC:POC, ratio of mineral-associated carbon to particulate organic carbon; Recalcitrant: labile ratio, ratio of recalcitrant to labile SOM components (see Methods). Al_d_+Fe_d_, dithionite-extractable Fe and Al oxides; CUE, carbon use efficiency; Pre, mean annual precipitation; Tem, mean annual temperature; AI, aridity index; NPP, net primary production; SOC, soil organic carbon; C:N, the ratio of soil carbon to nitrogen; F:B, the ratio of fungi to bacteria; τ, microbial biomass turnover rate; O:H, the ratio of oxidase to hydrolase.

**Fig. 3:**
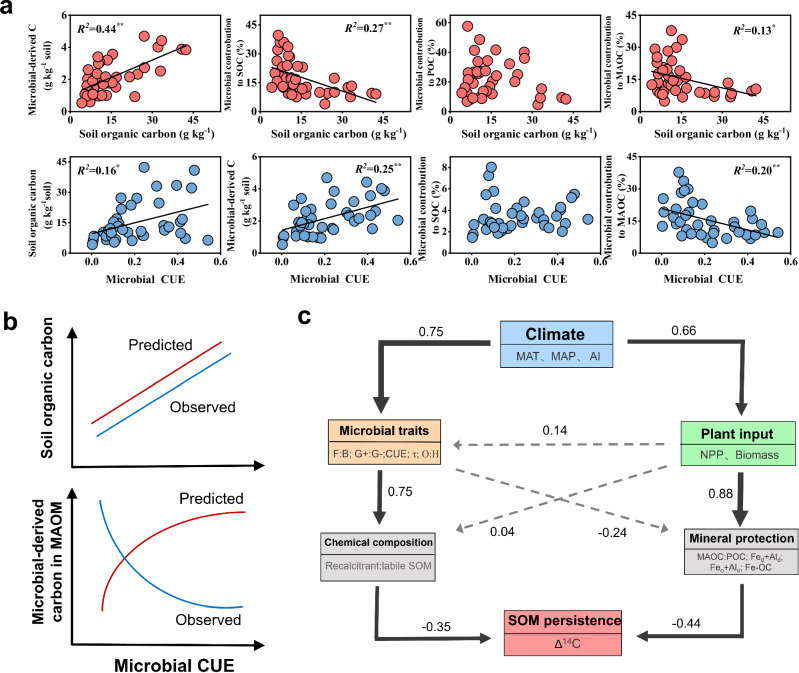
Pathways linking plant inputs, microbial processes, and soil organic matter (SOM) persistence. a, Relationships among microbial-derived carbon, soil organic carbon (SOC), and microbial carbon use efficiency (CUE). b, Schematic diagram illustrating the links between microbial CUE and SOC stabilization. c, Piecewise structural equation model showing the influences of biotic and abiotic factors on SOM persistence. Black solid lines indicate significant relationships (*P* < 0.05), whereas grey dashed lines indicate non-significant relationships (*P* > 0.05). Arrows indicate the directional influence of one variable upon another. The significance was determined at the *P* < 0.05 level. MAT, mean annual temperature; MAP, mean annual precipitation; AI, aridity index; NPP, net primary productivity; F:B, the ratio of fungi to bacteria; G + :G-, the ratio of Gram-positive to Gram-negative bacterial phospholipid fatty acids; τ, microbial biomass turnover rate; O:H, the ratio of oxidase to hydrolase; Al_d_+Fe_d_, reactive free oxides; Fe_o_+Al_o_, amorphous oxides; OC-Fe, ir_o_n-associated organic carbon; recalcitrant:labile ratio, ratio of recalcitrant to labile SOM components (see Methods).

Along the transect, soil Δ^14^C increased as SOC content rose *(P* < 0.01; Fig. 1d), indicating that SOC became younger and less persistent on average. The ratio of mineral-associated organic carbon (MAOC) to particulate organic carbon (POC), an indicator of physical protection via mineral association, significantly decreased as the SOC content increased (*P* < 0.05; Fig. 1c). Given that POM generally exhibits younger radiocarbon signatures than MAOM, the concurrent shift toward higher POM proportions contributes to the observed increase in bulk Δ^14^C, together indicating reduced SOM persistence in higher SOC soils. Concurrently, the correlation analysis showed that indices of mineral protection were closely associated with SOM physical composition and persistence, including MAOC:POC ratio and Δ^14^C (Fig. 2a). Specifically, the contents of dithionite-extractable Al and Fe oxides (Al_d_ and Fe_d_) and Fe-bound organic carbon (OC–Fe) increased significantly with SOC (*P* < 0.05), whereas the molar ratios of oxalate-extractable amorphous Al and Fe oxides (Alₒ and Feₒ) to SOC, as well as (Al_d_ + Fe_d_)/SOC, exhibited significant decreases (*P* < 0.01; Fig. S2). Correspondingly, the content of microbial-derived carbon (estimated using amino sugars, see Methods) increased with SOC (*P* < 0.05; Fig. 3a). However, its relative contribution to the total SOC pool significantly declined (*P* < 0.05). Furthermore, the ratio of microbial-derived carbon stored in the MAOM fraction compared to the POM fraction also decreased as SOC increased (*P* < 0.05; Fig. 3a; Fig. S3). Collectively, these results demonstrate that the higher SOC content is primarily associated with increased POM, alongside a declining proportion of mineral-stabilized microbial-derived carbon.

In assessing SOM chemical composition, we detected 167 types of compounds and grouped them into lipids, lignin derivatives, polysaccharides, proteins, nonprotein N-bearing compounds, aromatics, and phenolics (Fig. S4). Chemical diversity, indicated by the number of compounds (17–39 types), increased with the SOC content (Fig. 1c). The ratio of recalcitrant (e.g., lignin-derived phenols, long-chain alkyl compounds, and polyaromatic hydrocarbons) to labile compounds (e.g., polysaccharides and proteins) in SOM declined significantly with increasing SOC along the transect (Fig. 1c; see Methods for details), indicating reduced chemical recalcitrance. This pattern aligns with the observed increase in POM abundance. Overall, changes in SOM physical and chemical structure along the transect indicate that organic carbon in SOC-rich soils is younger and less physically and chemically stable than that in SOC-poor soils.

Microbial properties assessed along the transect included microbial biomass and community composition (based on phospholipid fatty acid (PLFA) analysis), microbial traits (CUE and biomass turnover rate (τ)), and extracellular enzyme activities. Based on PLFA, living soil microbial biomass, the ratio of fungi to bacteria, and the ratio of Gram-positive to Gram-negative bacteria all significantly increased with the aridity index (*P* < 0.05; Fig. S5). As important microbial physiological traits, microbial CUE and τ exhibited significantly increasing trends with aridity index (*P* < 0.05; Fig. S5). Further investigation of CUE (see Fig. S6 and explanation) indicated that higher CUE reflected enhanced microbial growth (*R*^2^ = 0.67) rather than decreased microbial respiration (*R*^2^ = 0.11). For extracellular enzymes, oxidase and hydrolase activities both increased with the aridity index, as did the ratio of oxidase to hydrolase activity (O:H; a measure of microbial substrate preference, with high ratios indicating greater allocation of resources to decompose more ‘recalcitrant’ organic compounds; Fig. S5; see Methods).

### Importance of abiotic and biotic factors for the physical and chemical structures of SOM

We performed regression analyses between SOM physical protection (MAOC:POC), chemical composition (recalcitrant: labile SOM compounds), SOM persistence (Δ^14^C) and climatic, soil and vegetation factors (Fig. 2a). The microbial properties (composition, enzyme activities, and CUE), mineral protection (Al_d_+Fe_d_), and SOM physical-chemical composition were highly correlated across the climate transect of the Tibetan Plateau (Fig. 2a). In particular, SOM physical stability (MAOC:POC) significantly declined as the ratio of fungi to bacteria increased (F:B; *R*^*2*^ = 0.17, *P* < 0.05), the ratio of oxidase to hydrolase enzymes (O:H ratio; *R*^*2*^ = 0.20), and CUE (*R*^*2*^ = 0.19), but increased with molar (Al_d_+Fed)/SOC (*R*^*2*^ = 0.46). For SOM chemical composition, the ratio of recalcitrant to labile components also showed significant negative relationships with the F:B ratio (*R*^*2*^ = 0.28), O:H ratio (*R*^*2*^ = 0.23) and CUE (*R*^*2*^ = 0.16), and a positive relationship with (Al_d_+Fed)/SOC (*R*^*2*^ = 0.12). Correspondingly, soil carbon persistence (Δ^14^C) was positively correlated with the F:B ratio (*R*^*2*^ = 0.41), O:H (*R*^*2*^ = 0.36), and microbial CUE (*R*^*2*^ = 0.30), but negatively correlated with the molar (Al_d_+Fe_d_)/SOC ratio (*R*^*2*^ = 0.10) (Fig. 2a). In contrast, the soil texture (clay and silt) content showed no significant relationship with SOC persistence (*P* > 0.05; Fig. 2b; Fig. S7).

We observed a significant positive correlation between microbial CUE and SOC, as well as microbial-derived carbon, across the transect (*R*^*2*^ = 0.16; Fig. 3a). However, the relative contribution of microbial-derived carbon to the total SOC pool did not increase with higher CUE. More specifically, its proportion within the mineral-associated organic matter (MAOM) fraction significantly declined (*R*^*2*^ = 0.20; Fig. 3a). This indicates that while more efficient microbial growth (high CUE) is associated with greater SOC storage and necromass production, it does not enhance the stabilization of this microbial residue into the long-term mineral-associated pool (Fig. 3b).

The Mantel test revealed that the physical composition of the SOM was strongly correlated with the aridity index, SOC, NPP and O:H ratio (Fig. 2b). The chemical composition of the SOM was highly related to F:B and O:H ratio. Random Forest modeling showed that NPP, F:B, pH, CUE, τ, O:H and soil C:N were the pivotal factors influencing SOM persistence (Δ^14^C), with increases in mean-square error (%IncMSE) of 15.7, 11.8, 8.9, 8.7, 6.6, 4.2 and 3.7, respectively. Taken together, plant inputs, soil properties and microbial properties were the most important predictors of SOM persistence along the alpine grassland transect.

Given strong correlations among climatic factors (precipitation and aridity index; Fig. 2b), plant inputs (NPP and biomass), microbial properties (community composition, enzyme activity, CUE, and τ; Fig. 2a, b; Table S2), and mineral protection (Fig. S2, S8 and S9), we employed a piecewise structural equation modeling (piecewise SEM) framework to disentangle the direct and indirect effects of these factors on SOM physical and chemical stability and SOM persistence (Fig. S10). The final model exhibited a satisfactory fit, as indicated by a non-significant Fisher’s C (*P* = 0.485), suggesting that the hypothesized causal structure was consistent with the observed data. The piecewise SEM revealed that climatic factors exerted significant effects on plant carbon inputs and microbial traits (*P* < 0.05). Plant carbon inputs had a strong positive effect on mineral protection, whereas microbial traits strongly influenced SOM chemical composition. In turn, both mineral protection and SOM chemical composition exerted significant negative effects on soil carbon persistence (Δ^14^C), indicating their roles in regulating carbon stabilization and turnover. These results indicate that plant and microbial pathways influence SOM persistence indirectly through distinct stabilization mechanisms. Collectively, these findings indicate that SOM persistence emerges from the combined influences of plant inputs, microbial regulation, and mineral-associated stabilization, highlighting the importance of multiple, interacting pathways in shaping long-term soil carbon dynamics.

### Global trends in SOC persistence

**Fig. 4:**
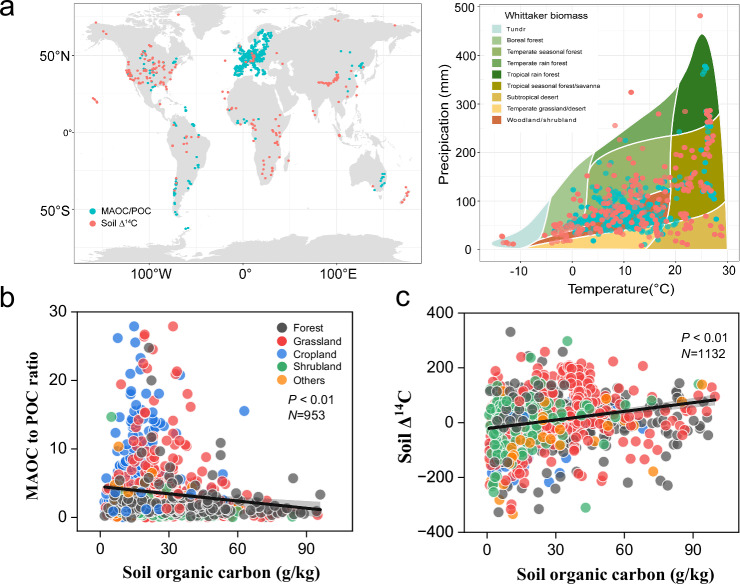
Global surface soil organic matter persistence trends. **a**, Global site distributions of soil fractionation and radiocarbon data. **b**, Relationships between the ratios of soil mineral-associated (MAOC) to particulate organic carbon (POC) and soil organic carbon concentrations. **c**, Relationships between soil radiocarbon (Δ^14^C) values and SOC concentrations. ^**^ indicates significant difference at the 0.01 level.

The global-scale analyses of the SOC fraction and radiocarbon databases were employed to test the generality of the emergent relationship between SOC content and its persistence, as reflected in its physical fractionation and radiocarbon signature (Fig. 4a). The MAOC:POC ratios significantly declined with increased SOC concentrations (*P* < 0.01; n = 953; Fig. 4b). This global pattern indicates that soils with higher organic carbon content contain a greater proportion of POM, which is largely plant-derived and less protected. The global radiocarbon data showed that the Δ^14^C values in surface soils significantly increased as SOC concentrations increased (*P* < 0.01; n = 1132; Fig. 4c). Since high Δ^14^C values indicate less stable SOC, these results consistently suggest that long-term SOM persistence declines as the SOC content increases. While these datasets lack the resolution to elucidate the underlying microbial mechanisms, they robustly confirm that the macroscopic pattern we observed regionally is a widespread phenomenon. Based on global analysis, we further mapped the global spatial pattern of surface soil organic matter persistence, indicated by the MAOC to POC ratios and soil Δ^14^C values (see Methods; Fig. S11, S12).

## Discussion

Our regional and global analyses consistently show that SOM persistence declines as SOC content increases, indicating that higher SOC content does not necessarily imply greater persistence. The global fractionation data, showing a decreasing MAOC:POC ratio with SOC content, indicate a systematic change in soil carbon pool composition towards faster-cycling forms. Similarly, higher Δ^14^C values in SOC-enriched soils indicate a greater contribution of recently cycled carbon or loss of older, stable carbon. Regional analyses provide a mechanistic explanation: while higher plant carbon inputs promote the accrual of soil carbon, they also facilitate a shift toward younger, faster-cycling carbon pools. This phenomenon suggests that the mechanisms driving SOC accumulation are not tightly coupled with those governing long-term persistence under high-input conditions. Elevated SOC in these ecosystems appears to be driven primarily by the expansion of active, labile fractions rather than the accumulation of “old”, stabilized carbon. The consistency of this pattern across spatial scales underscores its generality and highlights the need to reconsider how SOC stocks are interpreted in the context of long-term carbon sequestration.

We integrated soil radiocarbon (Δ^14^C) as a time-integrated indicator of SOM persistence with fraction-based and molecular-level analyses that provide mechanistic context for carbon stabilization and turnover. Along the SOC gradient, the observed “freshening” of bulk soil carbon (higher Δ^14^C) indicates a systematic shift toward younger carbon, reflecting an increased contribution of recently fixed inputs or an enhanced loss of older, more persistent carbon. This isotopic pattern coincides with shifts in SOM physical structure, particularly the increasing proportion of POM relative to MAOM. Because POM generally exhibits faster turnover rates and higher Δ^14^C values than mineral-bound fractions^25,26^, its increasing contribution is expected to elevate bulk Δ^14^C and reduce apparent soil carbon persistence. Molecular-level analyses further reinforce this interpretation. They reveal a shift toward more chemically labile compounds at the expense of structurally resistant components. In this context, radiocarbon signatures provide an additional, time-integrated perspective on SOM persistence, suggesting that the observed shifts in physical and chemical composition are accompanied by faster carbon turnover. Together, these independent lines of evidence indicate that SOC-rich soils are increasingly dominated by younger, more rapidly cycling carbon pools, offering a mechanistic explanation for the observed divergence between SOC content and persistence.

**Fig. 5:**
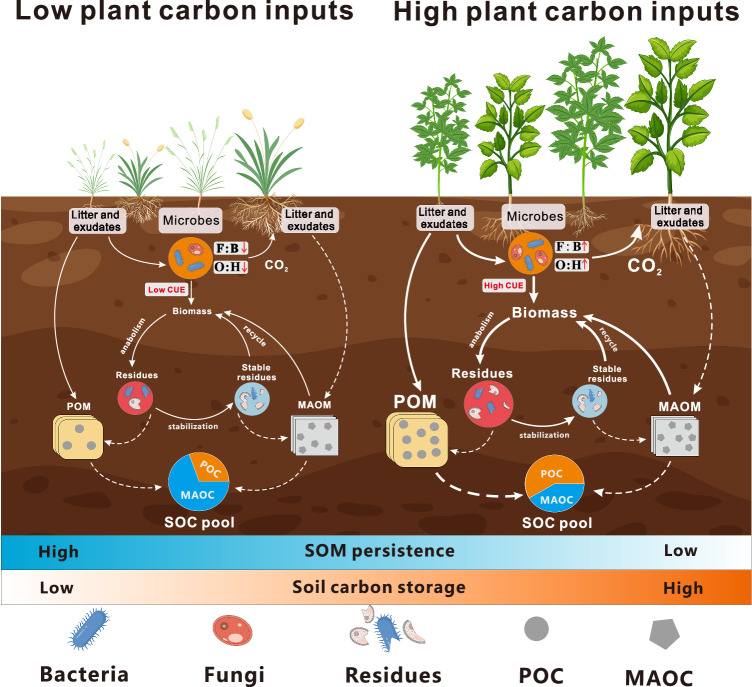
Conceptual framework illustrating how plant carbon inputs, mineral association, and microbial processing jointly regulate soil organic matter (SOM) persistence along the soil organic carbon (SOC) gradient. In SOC-poor soils, bulk SOM is characterized by lower Δ^14^C values (apparent older carbon) and relatively higher indices of mineral association and chemical resistance, indicating stronger apparent persistence. As SOC increases under higher plant inputs, bulk Δ^14^C increases and MAOC:POC declines, reflecting a growing contribution of faster-cycling particulate pools and more chemically labile compounds. Microbial traits (e.g., higher F:B ratios, higher oxidase-to-hydrolase ratios, and higher CUE) covary with this shift, consistent with intensified processing of fresh inputs and enhanced carbon throughput. Together, these patterns suggest that SOC accrual under high-input conditions can coincide with reduced persistence because gains in SOC are increasingly dominated by younger, rapidly cycling carbon, even though mineral-associated stabilization remains operative. Mineral-associated organic carbon to particulate organic carbon ratio (MAOC:POC); F:B, the ratio of fungi to bacteria; G + : G-, the ratio of Gram-positive bacteria to Gram-negative bacteria; CUE, carbon use efficiency; O:H, the ratio of oxidase to hydrolase; MAOM, mineral-associated organic matter; POM, particulate organic matter.

Our analyses identify NPP as an important predictor of SOM persistence. While higher plant carbon inputs promote SOC accumulation, they are associated with decreasing MAOC:POC ratios and lower recalcitrant to labile SOM ratios (Fig. S13). This suggests that additional carbon may predominantly accumulate in transient, chemically labile fractions, rather than mineral-associated pools. Consequently, elevated NPP is associated with faster carbon turnover rather than long-term retention. This shift aligns with patterns of enhanced microbial activity under high carbon-input conditions. Although declining leaf and soil C:N ratios suggest relatively greater nitrogen availability, the concurrent rise in the NPP:TN ratio shows that ecosystem nitrogen demand scales more rapidly than soil nitrogen stocks (Fig. S14), further supporting intensified microbial activity under relatively N-sufficient conditions. Increased inputs of recent plant-derived carbon create conditions that may promote priming-like effects^27^, potentially accelerating turnover of pre-existing SOM. This intensified carbon cycling is reflected in shifts in microbial traits. For example, higher oxidase-to-hydrolase ratios and increased fungal dominance (higher F:B ratios) indicate an enhanced capacity to decompose complex organic substrates^28,29^. Consistent with this intensified microbial processing, higher microbial CUE was associated with greater SOC accrual but also with faster microbial biomass turnover, suggesting that enhanced microbial production under high carbon input does not necessarily result in increased stabilization within mineral-associated pools (Fig. 5). Collectively, these findings indicate that under high carbon-input conditions, SOM persistence is increasingly influenced by plant inputs and microbially mediated turnover, contributing to reduced persistence with increasing SOC.

Our results support a conceptual model in which high plant carbon inputs accelerate SOC turnover and reduce its persistence. In this regime, increasing plant inputs promote the expansion of fast-cycling carbon pools and shift bulk SOM toward younger carbon. Although reactive Fe and Al oxides contribute to SOC stabilization, their relative influence becomes less dominant under high carbon-input conditions where recent plant inputs stimulate rapid carbon cycling. Consequently, SOC accumulation becomes increasingly decoupled from persistence, not because stabilization mechanisms are absent, but because intensified biological processing may limit long-term carbon retention. This perspective suggests that SOM persistence reflects the balance among plant inputs, mineral protection, and microbial processing, with important implications for projecting soil carbon–climate feedbacks.

Several limitations of this study should be acknowledged. First, soil samples collected along the regional transect were limited to surface soil. However, subsoil (below 0.3 m) stores more than half of the global SOC and exhibits substantial differences in carbon inputs, substrate availability, and microbial communities compared to topsoil^30,31^. Incorporating subsoil would provide a more comprehensive understanding of the patterns and related driving factors. Furthermore, this study only encompassed a limited variety of ecosystems owing to data availability, which can introduce uncertainties when extrapolating the findings to the global scale. Accounting for those under-studied ecosystems (e.g., forest and cropland) can further advance our understanding of microbe-driven SOC formation and stabilization. Lastly, while our findings highlight the crucial roles of plant carbon input and microbial properties in driving SOM persistence, other factors, such as soil moisture^32,33^, also have significant impacts on stable SOC preservation. These synergistic effects warrant further investigation in future studies.

In summary, our integrated analysis of a regional transect and global datasets provides compelling evidence that the persistence of SOM in surface soils declines as SOC content increases. Using soil radiocarbon as a time-integrated indicator of persistence, we show that SOC-rich soils are characterized by younger carbon ages and systematic shifts in SOM physical and chemical organization. These patterns reveal that SOC accumulation does not necessarily imply enhanced persistence. While increased plant inputs promote carbon accrual, they simultaneously shift the soil system toward younger, faster-cycling carbon pools. This decline in persistence is primarily driven by elevated NPP and associated microbial regulation, which accelerates carbon cycling. Our findings underscore the importance of incorporating plant inputs and microbial-mediated soil carbon persistence into process-based models to improve projections of soil carbon–climate feedbacks.

## Methods

### Site description and sampling

This study focused on soil samples obtained from a grassland-dominated transect located in the Tibetan Plateau (Fig. 1b). Spanning a length of 1500 km, the transect exhibits varying mean annual precipitation (ranging from 50 to 650 mm) and mean annual temperature (ranging from −3.6 to 2.3 °C) levels from east to west (http://www.worldclim.org). The transect spans grasslands from cold-arid to temperate climates, providing a natural gradient to examine plant and microbial pathways involved in SOM formation and stabilization. Owing to the pronounced aridity gradient observed along the transect (Fig. 1b), the aridity index was employed as an integrated indicator of climatic aridity (http://www.cgiarcsi.org/data/global-aridity-and-pet-database).

To encompass the wide environmental gradients, we established 40 sampling sites (spaced 40 to 50 km apart) in July 2020 in accordance with a standardized sampling method. At each site, five plots (10 m × 10 m) separated by distances of > 20 m were used as replicates, and three quadrats (1 m × 1 m) were placed along a diagonal line within each plot. Prior to sampling, surface litter was removed manually to ensure consistency across sites. Soil samples from the top 10 cm were collected using a soil auger (70 mm diameter) from the three quadrats and combined to form a composite sample for each plot. After homogenizing through a 2-mm sieve to remove plant roots, organic debris, and rocks, we randomly selected three out of five replicates at each site for laboratory analyses. The root samples were collected from soil cores (with a diameter of 70 mm), and they were cleaned and dried for belowground biomass measurements. For each quadrat, the aboveground biomass was determined by clipping the aboveground plants to ground level. NPP at each site was estimated from the NPP product provided by Moderate Resolution Imaging Spectroradiometer (http://modis.gsfc.nasa.gov), using the average from 2000 to 2020.

### Soil properties

The sieved soil samples were stored at 4 °C and transported to the laboratory. Subsamples of the whole soil were first weighed to determine fresh mass, oven-dried to measure soil moisture content, and thoroughly hand-picked to remove visible roots and organic debris. The dried soils were used for pH (H_2_O), total carbon and N analysis (Elementar Analysensysteme, Hanau, Germany), as well as for the determination of SOC, which was calculated as the difference between total carbon and soil inorganic carbon^34^. Soil inorganic carbon was measured after removing organic carbon by heating separate sample aliquots at 450 °C for 16 h, as well as density fractionation (see below). Portions of fresh soils were used to measure extractable nitrogen (NH_4_^+^-N and NO_3_^—^N)^35^, dissolved organic carbon, phospholipid fatty acids (PLFAs), and soil enzyme activity, as well as microbial CUE. The aboveground and belowground plant tissues weights were determined after being oven-dried at 65 °C for 48 h to constant weights. Tissues were ground and analyzed for total carbon and total N by a Vario Max CN analyzer (Elementar Analysensysteme, Hanau, Germany). Soil texture (sand, silt, and clay) was measured using the wet-sieving technique.

### SOM Physical Partitioning

Mineral-associated protection of SOM was inferred from the ratio of MAOC to POC, determined using physical fractionation^36,37^. Briefly, each 30-g subsample of air-dried soil was shaken with a 0.5% sodium hexametaphosphate solution on a reciprocal shaker for 18 h to disperse aggregates. The dispersed soil samples were then rinsed onto a 53-μm sieve with deionized water to separate and collect POM (53–2000 μm). Each soil slurry that passed through the 53-μm sieve was then transferred and dried at 50 °C to collect the MAOM fraction (<53 μm). Both POM and MAOM fractions were subsequently weighed and ground. Their respective concentrations of carbon and N were determined and MAOC and POC (g C kg⁻¹ soil) were calculated.

### Mineral protection capacity

To quantify mineral protection capacity, we measured Al/Fe-oxides, including free (Al_d_/Fe_d_), amorphous (Al_o_/Fe_o_), and complexed (Al_p_/Fe_p_) forms, using established chemical extraction methods. Al_d_/Fe_d_ was extracted using citrate-bicarbonate-dithionite^38^. The Al_o_/Fe_o_ and Al_p_/Fe_p_ fractions were isolated using 0.2 M ammonium oxalate (pH 3.0) and 0.2 M sodium pyrophosphate solutions, respectively^16^. The content of iron-associated organic carbon (OC-Fe) and its proportion to total organic carbon (fOC-Fe) were determined to evaluate Fe-mediated stabilization. Furthermore, the molar ratios of SOC to reactive Fe/Al oxides were determined to evaluate the capacity of the mineral matrix for SOC stabilization^39^.

### Chemical recalcitrance

SOM chemical characteristics of bulk soil were assessed using pyrolysis-gas chromatography–mass spectrometry (Py-GC/MS). Because of the high costs of Py-GC/MS analyses, subsamples of three replicates were mixed as one composite sample. Compound peaks were analyzed and identified using the Automated Mass Spectral Deconvolution and TurboMass software for GC/MS (version 6.1), the National Institute of Standards and Technology (NIST) compound library and previously published literature. Organic matter compounds were quantified as the percentage relative abundances of the total sample peak areas and classified based on their probable origin: alkyl compound, polysaccharide―derived compound, lignin, phenol, chitin, N compound, aromatic, or polyaromatic. Compounds that could potentially have multiple origins were classified as ‘unspecified’.

Based on their molecular structure and established biochemical persistence, the identified compounds were tionally classified into two distinct pools on:(1) a labile SOM pool, comprising compounds that are readily metabolized by a broad range of soil microorganisms via hydrolytic enzymes, including polysaccharides (marked by furans, furfurals, and pyran derivatives) and proteins (indicated by pyrroles, pyridines, and indoles); and (2) a recalcitrant SOM pool, encompassing compounds that require specific oxidative enzymes for degradation, including lignin-derived phenols, long-chain alkyl compounds, and polyaromatic hydrocarbons^14,40–42^. The molecular diversity of the SOM was calculated based on molecular richness and the Shannon-Wiener index as a measure of compositional complexity^43^. Given that the stability of SOM is governed by its molecular structure and accessibility, the ratio of recalcitrant to labile compounds was used as an indicator of the relative stability and biogeochemical persistence of the SOM.

### Microbial-derived SOC analysis

Microbial-derived SOC (hereafter referred to as microbial necromass carbon) was estimated from the amount of extracted amino sugars^44^ using a slightly modified procedure^45^. Measurements were performed on bulk soil as well as on the isolated MAOM and POM fractions. Amino sugars primarily originate from microbial cell walls and are widely used as biomarkers of microbial residues^46^. As such, they serve as important biomarkers for microbial residues. Conversion factors have been established that link the most common amino sugars, glucosamine and muramic acid, to their relative sources from fungal and bacterial necromass^47^. Briefly, each 1-g soil sample was hydrolyzed with 10 mL of 6 M HCl at 105 °C for 6 h. After hydrolysis, the samples were mixed thoroughly and allowed to cool to room temperature before being filtered. Subsequently, 0.5 mL of the filtrate was evaporated using nitrogen gas at 40–45 °C to remove HCl. The dried residues were dissolved in 0.5 mL of deionized water, dried by nitrogen gas again, re-dissolved in 2 mL of deionized water, and stored at −20 °C before analysis. The extracted amino sugars were derivatized using O-phthaldialdehyd, and the derivatives were separated using a high-performance liquid chromatograph (Dionex Ultimate3000, Thermo Fisher Scientific, USA) equipped with an octadecylsilylated silicagel column (Acclaim120 C18; 4.6 mm × 150 mm, 3 μm; Thermo Fisher Scientific). Amino sugars were detected using a fluorescence detector with an excitation wavelength of 330 nm and an emission wavelength of 445 nm. The identification and quantification of amino sugars were achieved by comparing the chromatograms of the samples with those of standard solutions containing mixed amino sugars. Total amino sugars were calculated as the sum of muramic acid, glucosamine and galactosamine. Fungal- and bacterial-derived carbon were estimated from amino sugar biomarkers and used to represent fungal and bacterial necromass, respectively^47^(Appendix S1).

### Soil microbial composition, microbial CUE, and enzyme activities

Phospholipid fatty acids (PLFAs) are components of microbial cell membranes. Because of the rapid recycling of phosphorus in soils, PLFAs are commonly assumed to represent the living microbial community^48^, with specific compounds serving as biomarkers for specific kinds of bacteria or fungi. PLFAs were measured to determine microbial community composition using the standard procedures and nomenclature^49^. The sum of PLFAs was calculated as an indicator of the total living microbial biomass^50^. The PLFAs specific to fungi (18:2ω6,9c), Gram-negative bacteria (16:1ω7c, cy17:0, 18:1ω7c and cy19:0), and Gram-positive bacteria (i14:0, i15:0, a15:0, i16:0, i17:0 and a17:0) were independently quantified^48^. The bacterial PLFAs were calculated as the sum of Gram-negative and Gram-positive bacterial PLFAs. The composition of the microbial community was characterized by determining the ratios of fungal to bacterial phospholipid fatty acids (F:B) and of Gram-negative to Gram-positive bacterial PLFAs (Gram-negative: Gram-positive).

Soil microbial carbon use efficiency (CUE), a measure of the amount of carbon used to grow microbial cells versus that lost via respiration or exudation, was determined using the ^18^O-H_2_O tracer method^51^. For this assay, soil water content was adjusted to 60% of the water holding capacity (WHC) and then pre-incubated at 15 °C for 7 d prior to the CUE measurement. Briefly, duplicate aliquots of 600 mg of each preincubated soil (24 h) were placed into 2-ml brown chromatographic vials. For one replicate, the ^18^O-H_2_O (97.0 at% ^18^O) was added to adjust the ^18^O content of the soil water to 20.0 at% ^18^O, whereas non-labeled water was added to the other replicate to serve as a natural ^18^O abundance control. The vials containing the samples were then transferred to 20-ml headspace bottles, sealed, and flushed with CO_2_-free air to obtain headspace concentrations of CO_2_ close to 0 ppm. Five blank bottles without soil were also prepared and subjected to the same procedures to serve as negative controls in the CO_2_ concentration analysis. The samples were incubated for 24 h at 15°C, which is similar to the mean growing season temperature across the transect. After 24 h of incubation, 10 ml gas sample was collected from each bottle with a syringe and the CO_2_ concentration was determined immediately using a gas chromatograph system (GC-7890B; Agilent Technologies). Following the gas sampling, the brown vials containing the soil were retrieved, capped, frozen immediately in a lyophilizer, and subsequently stored at −80 °C until DNA extraction. Total soil DNA was extracted from the frozen samples using a DNA extraction kit (MoBio, Powersoil), following the manufacturer’s procedures. The DNA concentration was determined using the PicoGreen fluorescence assay (Quant-Ittm PicoGreen dsDNA Reagent, Thermo Fisher) and a microplate spectrophotometer (Infinite® M200, Tecan, Austria). The remaining DNA extract was dried in a silver capsule at 45 °C for 5 h to remove any residual water. Subsequently, the ^18^O abundance and the total O content were measured using an IRMS-TC/EA (Thermo Scientific, TX, USA). The microbial biomass carbon (MBC) was determined using the CHCl_3_ fumigation extraction method^52^. Microbial CUE was calculated as microbial growth divided by the sum of growth and respiration (Appendix S2).

In total, the activities of five soil carbon-degrading enzymes, β−1,4-glucosidase, β−1,4-xylosidase, cellobiohydrolase, phenol oxidase, and peroxidase, were measured^53,54^. Enzymes with distinct capacities to handle steric hindrance might participate in polymer degradation processes. Hydrolases (H), including β−1,4-glucosidase, β−1, 4-xylosidase, and cellobiohydrolase, mainly catalyze the hydrolysis of cellulose and hemicellulose, whereas oxidases(O), including phenol oxidase and peroxidase, promote the degradation of phenolic-containing recalcitrant compounds^55^. The O:H ratio serves as a reliable indicator of microbial substrate preference, and high ratios signify relatively greater allocations of resources towards decomposing chemically recalcitrant or complex organic carbons^56^, i.e. those requiring higher amount of energy investment to decompose.

### Radiocarbon (^14^C) measurements

As a valuable tool for exploring SOM dynamics on different timescales, soil Δ^14^C has been widely applied to characterize SOM persistence. Generally, lower Δ^14^C value generally indicates older and more persistent soil carbon^10^. In brief, soil samples were first decalcified using 1-M hydrochloric acid, freeze dried, and combusted to obtain CO_2_ gas. The evolved purified CO_2_ was then frozen in a reduction tube and reduced to graphite under H_2_ atmosphere at 550 °C. The graphite was measured using an accelerator mass spectrometer at the Max Planck Institute for Biogeochemistry in Jena, Germany. The Δ^14^C data was calculated as the per mil deviation in ^14^C/^12^C ratio from an absolute standard^57^.

### Global soil MAOC, POC and radiocarbon data synthesis

This global analysis was designed to assess the generality of the relationship between SOC content and its persistence, rather than to elucidate the underlying microbial mechanisms. To explore the correlations between SOC content and MAOC:POC across global biomes, data from globally distributed topsoil (0–30 cm) were synthesized^58^. POC was calculated as SOC minus MAOC when not explicitly reported. To characterize SOM persistence globally, we extracted topsoil (0–30 cm) Δ^14^C data from the International Soil Radiocarbon Database^59^. Because we focused on soil fractionation patterns in mineral soils, organic-rich soils with total organic carbon levels greater than 100 g C kg^−1^ (e.g., peatlands) were excluded from the analysis. To minimize the influence of extreme values, we identified outliers as the measurements exceeding −3 or +3 standard deviations from the population mean for global soil fractions and the Δ^14^C dataset following standard outlier removal practices. Ultimately, 953 and 1,132 data points were extracted from the Global Soil Carbon Fractionation Database and the International Soil Radiocarbon Database, respectively.

### Mapping global soil organic matter persistence

To predict SOM persistence globally, we produced maps of the MAOC:POC and soil Δ^14^C across the globe at the resolution of 0.0083° × 0.0083° using Random Forest models implemented in the R package randomForest. The ‘RandomForest’ library of R-core (https://www.r-project.org/) was applied to predict SOM persistence from the following predictors: mean annual temperature, mean annual precipitation, aridity index, elevation, NPP, SOC, pH, soil total nitrogen content, soil C:N ratio, sand content, silt content, and clay content. Long-term mean annual temperature and mean annual precipitation were obtained from the WorldClim datasets (https://worldclim.org/). Elevation was obtained from Global Version Elevation (https://globalmaps.github.io/el.html). Corresponding soil data (SOC, pH, soil total nitrogen content, soil C:N ratio, sand content, silt content, and clay content) were obtained from The Harmonized World Soil Database (HWSD) version 2.0 (https://gaez.fao.org/pages/hwsd). Due to data deficiency, permanent snow and ice, barren land, and water bodies were removed based on the MODIS land cover map.

### Statistical analysis

To identify the key drivers of SOC physical stability and chemical recalcitrance, we employed a multi-method analytical approach using R version 4.2.2. A linear mixed model with fixed effects of environmental and biotic factors and sampling site as a random intercept was fitted along the transect. Mantel tests and Random Forest analyses were further performed to evaluate the relationships between microbial traits, soil properties, and SOC persistence indicators. Structural equation modeling (SEM) was conducted using a piecewise framework implemented in the R package piecewiseSEM. This approach represents hypothesized causal relationships as a set of linked linear models and evaluates overall model fit using Fisher’s C statistic derived from tests of directed separation. An a priori model was first specified based on ecological hypotheses (Fig. S10). Model refinement was conducted by iteratively removing unsupported paths with non-significant coefficients, while ensuring consistency with directed separation tests and overall model fit.

### Reporting summary

Further information on research design is available in the Nature Portfolio Reporting Summary linked to this article.

## Supplementary information

Supplementary Information

Reporting Summary

Transparent Peer Review file

## Data Availability

The datasets supporting the findings are available in the figshare repository (10.6084/m9.figshare.28636262).
