# Supplementary material for: Declines in organic matter persistence with increased soil carbon
Source: Nat Commun. 2026 Jul 7;17:8521. doi: 10.1038/s41467-026-75185-4 (PMC13482611; doi:10.1038/s41467-026-75185-4)
Supplement: Supplementary file 1 [file 41467_2026_75185_MOESM1_ESM.pdf]

SUPPLEMENTARY MATERIALS

Declines in organic matter persistence with increased soil carbon

Guang Zhao et al.

Appendix S1: Microbial-derived carbon calculations

Appendix S2: Soil microbial carbon use efficiency calculations

Supplementary Table 1. Climate, plant and soil properties of 40 sample sites

Supplementary Table 2 Principal components analysis (PCA) results on Climate, soil properties, soil microbial traits, soil organic matter stability.

Supplementary Fig. 1 Changes in precipitation, mean air temperature, aridity index and soil moisture along longitude.

Supplementary Fig. 2 Correlations between iron-aluminum oxides and SOC content.

Supplementary Fig. 3 Correlations between amino sugar, amino sugar in POM and MAOM and SOC content.

Supplementary Fig. 4 Principal component analysis of chemical compositions in soil organic matter.

Supplementary Fig. 5 Relationships between biotic, abiotic variables and aridity index.

Supplementary Fig. 6 Relationships between qGrowth, qCO_2 and carbon use efficiency.

Supplementary Fig. 7 Relative weight ratio of soil density fractionation (a) and soil texture (b)

Supplementary Fig. 8 Correlations between iron-aluminum oxides and the ratio of MAOC to POC.

Supplementary Fig. 9 Correlations between iron-aluminum oxides and soil carbon persistence ($\Delta^{14}C$).

Supplementary Fig. 10 A priori model for structural equation modeling (SEM) analysis of soil organic matter persistence along the grassland transect.

Supplementary Fig. 11 Relative importance of environmental variables in influencing MAOC to POC ratio (**a**) and $\Delta^{14}C$ (**b**) revealed by random forest

model.

Supplementary Fig.12 Global spatial pattern of surface soil organic matter persistence, indicated by the MAOC to POC ratio (**a**) and soil $\Delta^{14}C$ values (**b**) across the globe at the resolution of 0.0083°. MAOC, mineral-associated organic carbon; POC, particulate organic carbon.

Supplementary Fig. 13 Relationships of MAOC:POC ratio and recalcitrant to labile SOM ratio with NPP.

Supplementary Fig. 14 Stoichiometry of plant and soil along the transect.

Appendix S1: Microbial-derived carbon calculations

Fungal-derived carbon and bacterial-derived carbon are commonly used as biomarkers of fungal residues and bacterial residues. Bacterial-derived carbon was calculated as:

$$Bacterial\ carbon\ (mg{\cdot}g^{-1}) = MurN\ (mg{\cdot}g^{-1}) \times 45 \quad (1)$$

where 45 represents the conversion value of MurN (muramic acid; mg·g^{-1}) to bacterial carbon (Engelking *et al.*, 2007).

Assuming MurN and GluN (glucosamine) are present at a 1:2 molar ratio in bacterial cells, fungal-derived carbon was calculated as:

$$Fungal\ carbon\ \ (mg{\cdot}g^{-1}) = (GluN - 2 \times MurN) \times 9 \quad (2)$$

where 9 represents the conversion coefficient of fungal-derived GluN (mg·g^{-1}) to fungal carbon (Engelking *et al.*, 2007).

Microbial-derived carbon is calculated as the sum of bacterial carbon and fungal carbon.

Appendix S2: Soil microbial carbon use efficiency calculations

Soil microbial carbon use efficiency was calculated according to Zheng et al. (2019):

$$DNA_{produced} = O_{total} * \frac{at\%_{excess}}{100} * \frac{100}{at\%_{label}} * \frac{100}{31.21} \quad (3)$$

Where $DNA_{produced}$ refers to the total DNA produced (μg) during the 24-hour incubation period, and O_{total} is the total O content (μg) of the dried DNA extract, $at\%_{excess}$ is the difference between the abundance of ^{18}O in samples with and without $H_2{}^{18}O$; $at\%_{label}$ is the ^{18}O enrichment of soil water, and 31.21% is the average weight% of O in DNA.

$$C_{growth} = \frac{f_{DNA} * DNA_{produced} * 1000}{DW * t} \quad (4)$$

Where C_{growth} is the microbial growth rates (ng C·g^{-1}·dry soil), and f_{DNA} is the ratio of soil MBC (μg g^{-1} DW) to soil DNA content (μg g^{-1} DW), and DW is the dry mass of soil for DNA extraction in grams, and t is incubation time (hr).

$$C_{respiration} = \frac{D_{CO_2}}{DW * \mathrm{t}} * \frac{p * n}{R * T} * V_{hs} * 1000 \quad (5)$$

Where $C_{respiration}$ is microbial respiration rate (ng C·g^{-1}·h^{-1}), D_{CO2} is the increase in CO_2 concentration (ppm) produced during the incubation period, p is the atmosphere pressure (kPa), n is the molecular mass of the element C (12.01 g mol^{-1}), R is the ideal gas constant (8.314 J mol^{-1} K^{-1}), T is the incubation temperature (295.15 K), V_{hs} is the volume (L) of the headspace volume.

$$CUE = \frac{C_{growth}}{C_{growth} + C_{respiration}} \quad (6)$$

Microbial biomass turnover time (d; $t_{turnover}$) was calculated as follows:

$$t_{turnover} = \frac{DNA_{amount}}{DNA_{produced} * t/24} \quad (7)$$

where DNA_{amount} is the DNA content in each soil quantified by Picogreen assay, and t is incubation time in hours.

Microbial biomass-based growth rates (*qGrowth*) and metabolic quotients (qCO_2) can be estimated as follows:

$$qGrowth=\frac{C_{growth}}{MBC} \tag{8}$$

$$qCO_2=\frac{C_{respiration}}{MBC} \tag{9}$$

Supplementary Table 1 Climate, plant and soil properties of 40 sample sites

No.	Latitude (°N)	Longitude (°E)	MAP (mm)	MAT (°C)	Plant biomass (g m^{-2})	NPP (g m^{-2})	pH	TN (%)	Soil C:N	DOC (mg kg^{-1})	AN (mg kg^{-1})
1	31.93	93.98	654.4	1.37	4234.72	302.86	7.34	0.37	12.6	80.9	69.6
2	31.95	93.92	645.5	1.85	2392.19	371.30	6.88	0.25	10.5	42.2	52.4
3	31.75	93.75	657.4	2.26	2054.02	367.37	6.70	0.31	11.6	53.6	35.2
4	31.92	93.09	604.0	-0.33	4936.07	383.40	6.66	0.26	15.1	59.4	15.6
5	31.67	92.34	526.4	-0.52	3349.69	369.58	6.80	0.18	14.9	50.6	34.5
6	31.40	90.31	503.1	0.56	2902.85	282.01	6.86	0.29	13.6	46.2	27.8
7	31.37	90.43	497.3	0.81	1904.43	150.00	7.16	0.19	12.8	38.5	29.0
8	31.40	90.79	492.5	0.27	411.34	37.33	8.29	0.07	25.2	28.3	11.7
9	31.61	90.72	500.8	-1.07	487.78	58.13	8.09	0.08	9.7	29.9	18.9
10	31.64	92.02	495.5	-0.94	270.06	57.70	7.64	0.08	10.2	34.1	17.3
11	31.63	89.58	490.4	1.38	134.39	65.55	8.06	0.10	13.6	36.1	21.2
12	31.52	89.24	483.1	1.56	85.61	45.41	7.99	0.03	37.5	24.0	11.7
13	31.49	88.91	469.9	2.27	206.33	70.09	8.57	0.09	37.8	28.4	15.2
14	31.69	88.52	435.2	2.00	164.85	60.00	8.13	0.11	25.9	32.1	18.5
15	31.84	88.34	397.4	1.54	126.50	44.37	8.52	0.05	25.0	28.3	8.7
16	31.83	88.06	365.6	0.75	266.24	46.41	8.31	0.17	11.1	44.7	23.0
17	31.80	87.48	306.7	2.06	369.04	46.69	8.69	0.07	26.0	26.8	8.3
18	31.83	87.16	264.2	1.37	420.97	44.69	8.33	0.11	12.8	34.0	22.3
19	31.94	86.88	234.8	1.84	319.34	52.66	8.45	0.11	17.0	40.2	17.3
20	31.92	86.46	190.6	0.28	220.09	51.29	8.77	0.08	14.1	24.9	9.4
21	31.86	86.10	166.1	-0.74	285.64	46.55	8.84	0.11	26.8	31.6	20.6
22	31.94	85.80	142.7	-2.41	459.23	45.27	8.91	0.09	51.6	43.3	26.2
23	32.02	85.45	123.6	-1.34	202.02	44.74	8.66	0.08	48.7	22.8	11.6
24	32.31	83.76	85.8	1.68	259.11	42.22	8.64	0.10	44.9	33.8	12.3
25	32.36	83.61	83.7	1.30	214.74	56.95	8.51	0.08	23.7	22.0	13.3
26	31.99	84.83	119.1	0.81	385.63	49.44	8.65	0.06	18.0	30.7	14.0
27	32.22	84.47	103.8	1.30	172.24	43.82	9.22	0.06	33.4	28.7	3.8
28	32.25	84.27	93.2	0.76	286.80	45.48	8.59	0.09	16.6	32.1	12.9
29	32.41	83.40	78.4	-0.11	219.15	50.00	8.65	0.10	9.0	28.4	15.7
30	32.44	83.15	79.9	0.66	380.65	49.15	8.60	0.11	14.3	21.9	12.3
31	32.42	82.81	77.8	0.94	110.83	37.80	8.57	0.09	9.8	23.3	8.7
32	32.49	82.42	84.6	0.27	183.63	51.26	8.43	0.09	9.5	17.1	11.4
33	32.24	82.14	93.7	-0.06	199.61	27.48	8.32	0.07	19.9	15.8	6.6
34	32.07	81.82	116.0	-0.37	611.85	31.74	8.20	0.08	21.5	28.6	12.2
35	32.17	81.60	103.4	-3.61	226.15	38.27	8.16	0.16	28.6	19.2	13.3
36	32.27	81.25	106.2	-0.47	82.24	24.72	8.55	0.06	14.2	13.3	3.4
37	32.39	80.79	97.35	-0.16	158.87	50.01	8.95	0.03	27.0	15.7	3.4
38	32.41	80.28	104.0	-1.28	73.25	31.11	8.29	0.06	7.4	10.7	2.9
39	32.72	79.83	83.1	-2.62	144.20	29.01	8.68	0.06	20.5	12.9	4.3
40	33.21	79.81	54.9	-0.63	95.40	23.92	8.66	0.05	22.0	24.5	5.3

Supplementary Table 2 Principal components analysis (PCA) results on climate, plant input, soil microbial traits, mineral protection, and chemical stability. Asterisks in the table indicate the strength of linear relationships between variable and PC1(*, P < 0.05; **, P < 0.01).

Factors	PC1
**Climate**	
MAT	-0.55**
MAP	0.98**
AI	0.96**
Cumulative (%)	72.79
**Plant input**	
NPP	0.98**
Biomass	0.98**
Cumulative (%)	96.16
**Microbial traits**	
F:B	-0.62**
G+:G-	-0.74**
CUE	-0.72**
τ	-0.75**
O:H	-0.81**
Cumulative (%)	51.71
**Mineral protection**	
MAOC:POC	0.15
Al_d+Fe_d	-0.88**
Fe_o+Al_o	-0.96**
OC-Fe	-0.87**
Cumulative (%)	62.1

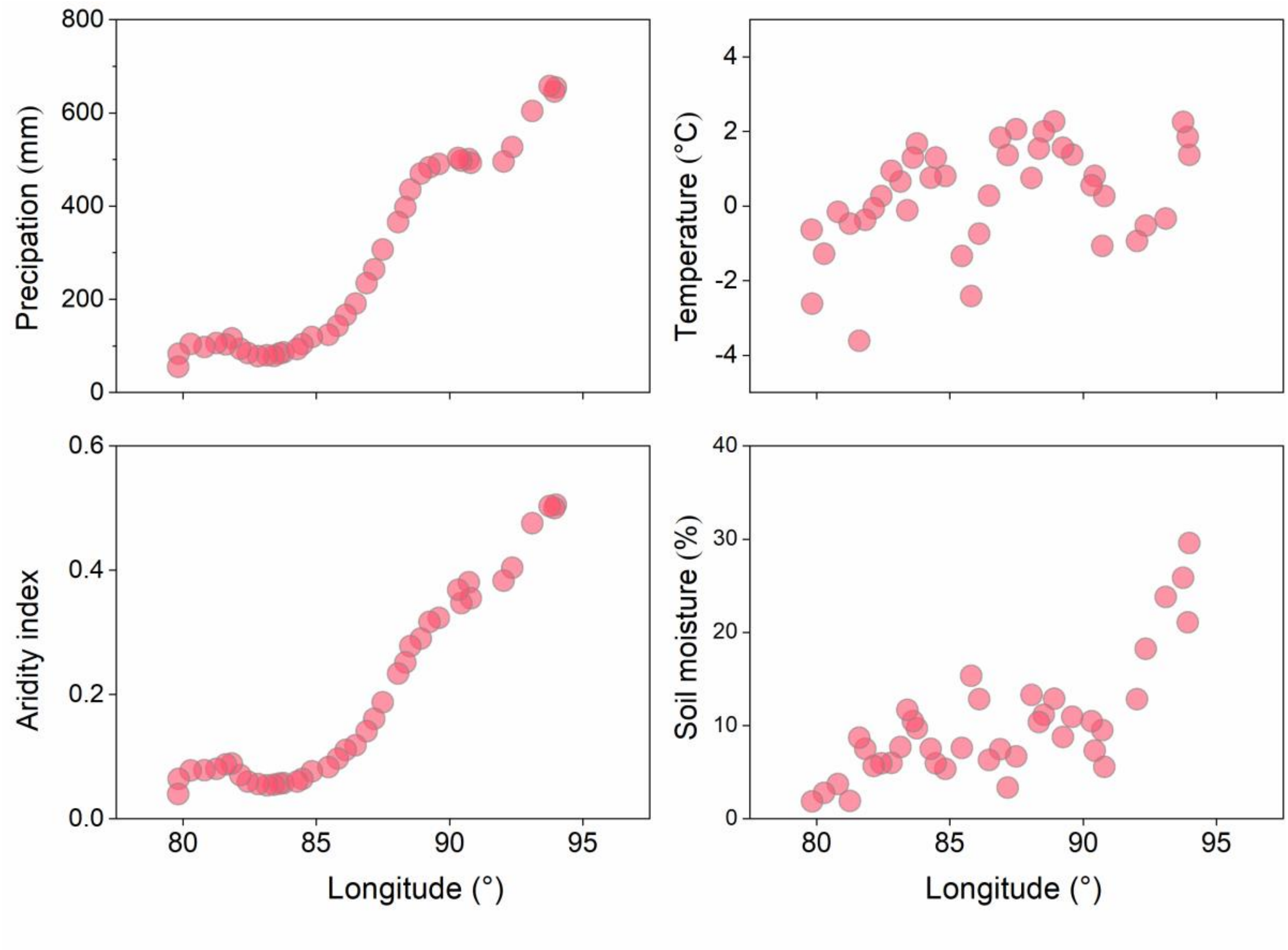

Supplementary Fig. 1 Changes in precipitation, mean air temperature, aridity index and soil moisture along longitude.

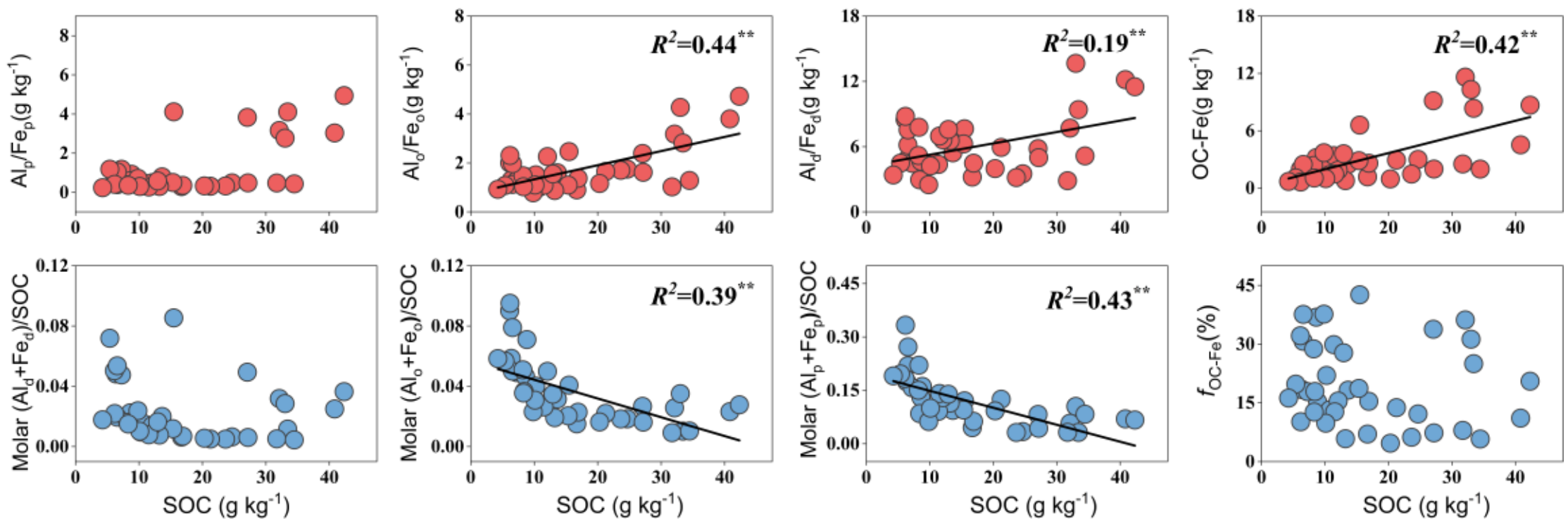

Supplementary Fig. 2 Correlations between iron-aluminum oxides and SOC content.

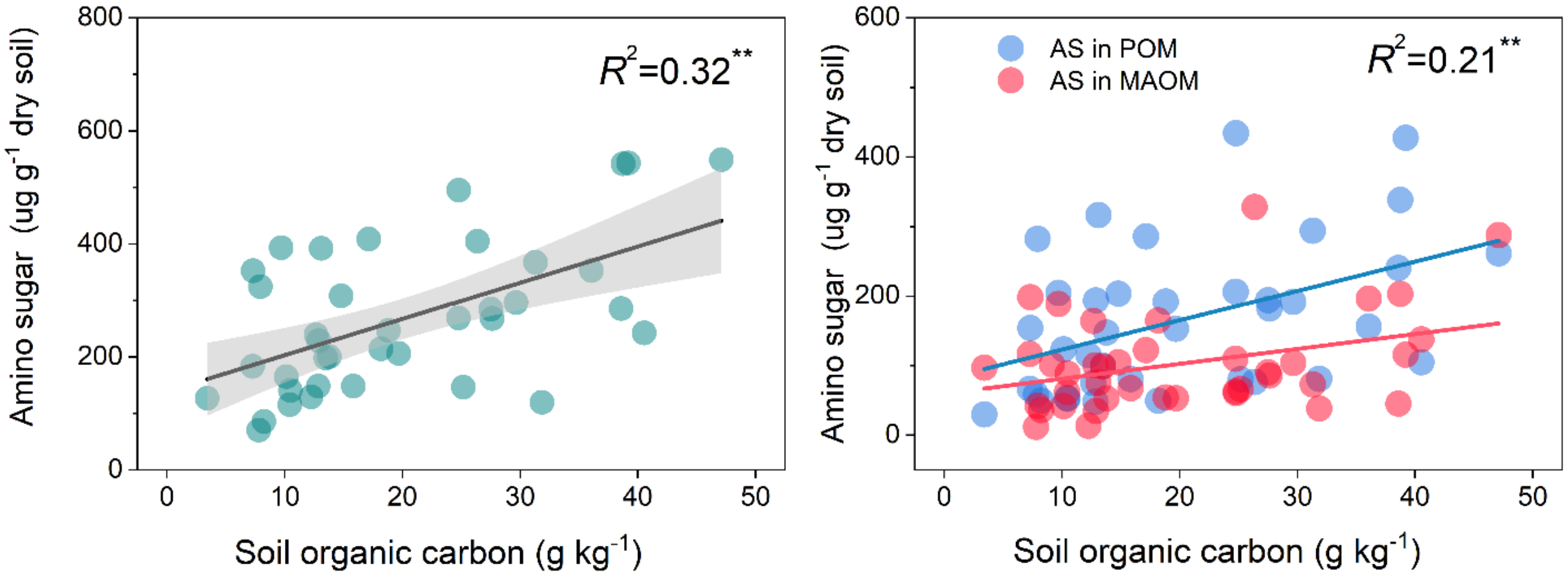

Supplementary Fig. 3 Correlations between amino sugars in bulk soil, POM, and MAOM and SOC content.

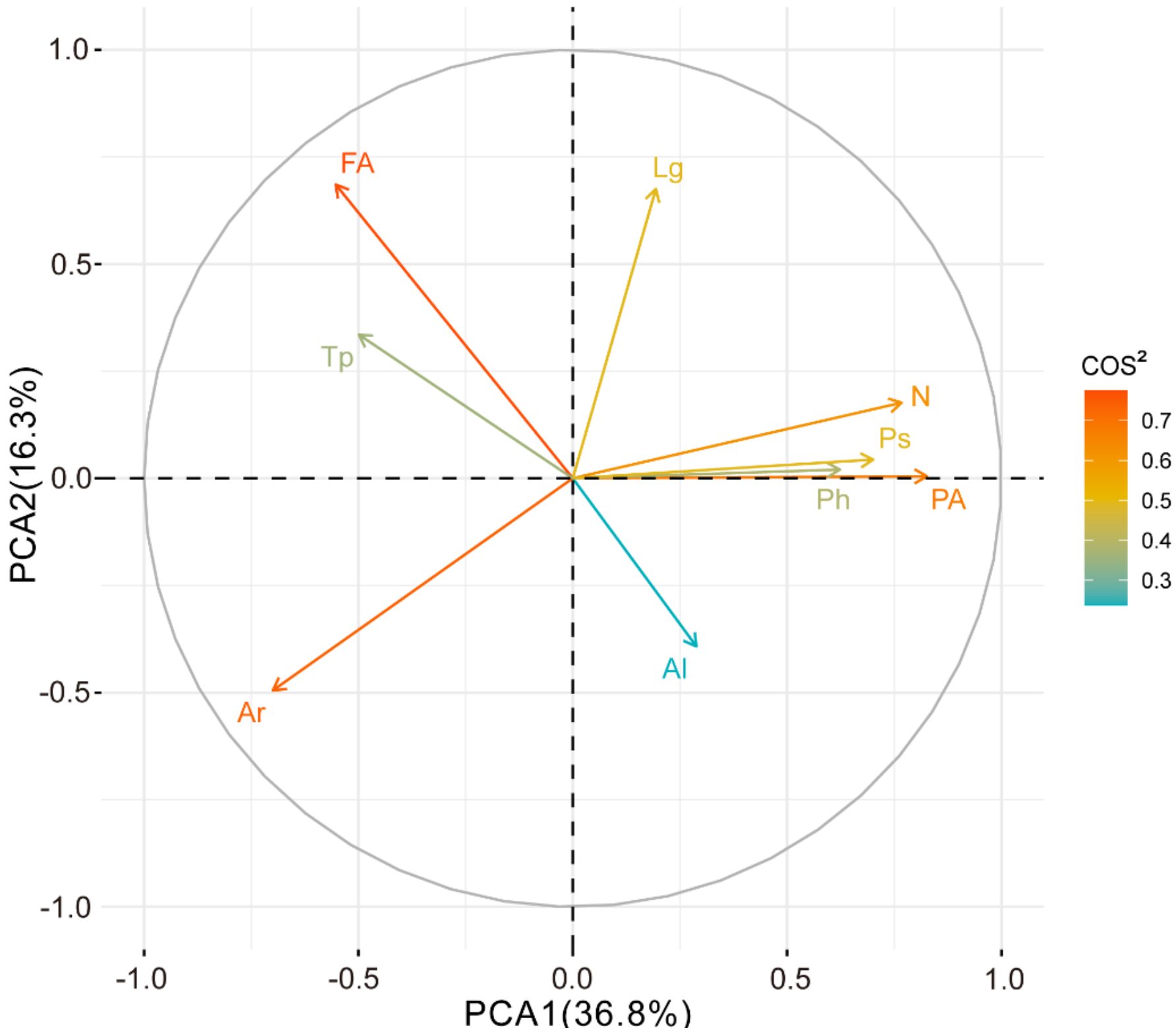

Supplementary Fig. 4 Principal component analysis of chemical compositions in soil organic matter. Ar, Aromatics; Fatty acids, FA; Lg, Lignin; N, N compounds; PA, Polyaromatic; Phenol, Ph; Ps, Polysaccharides; Al, Alkyls.

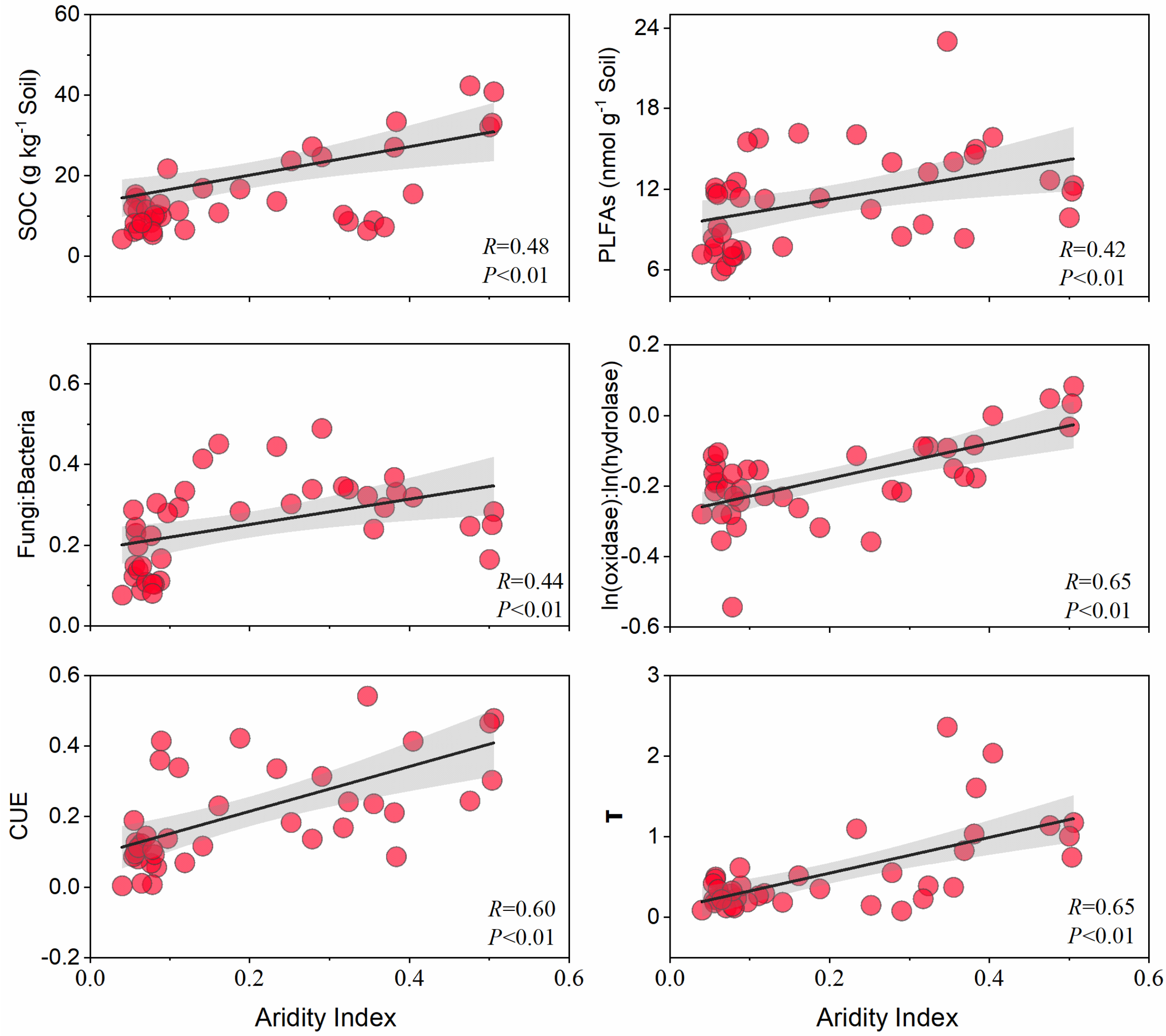

Supplementary Fig. 5 Relationships between biotic, abiotic variables and aridity index.

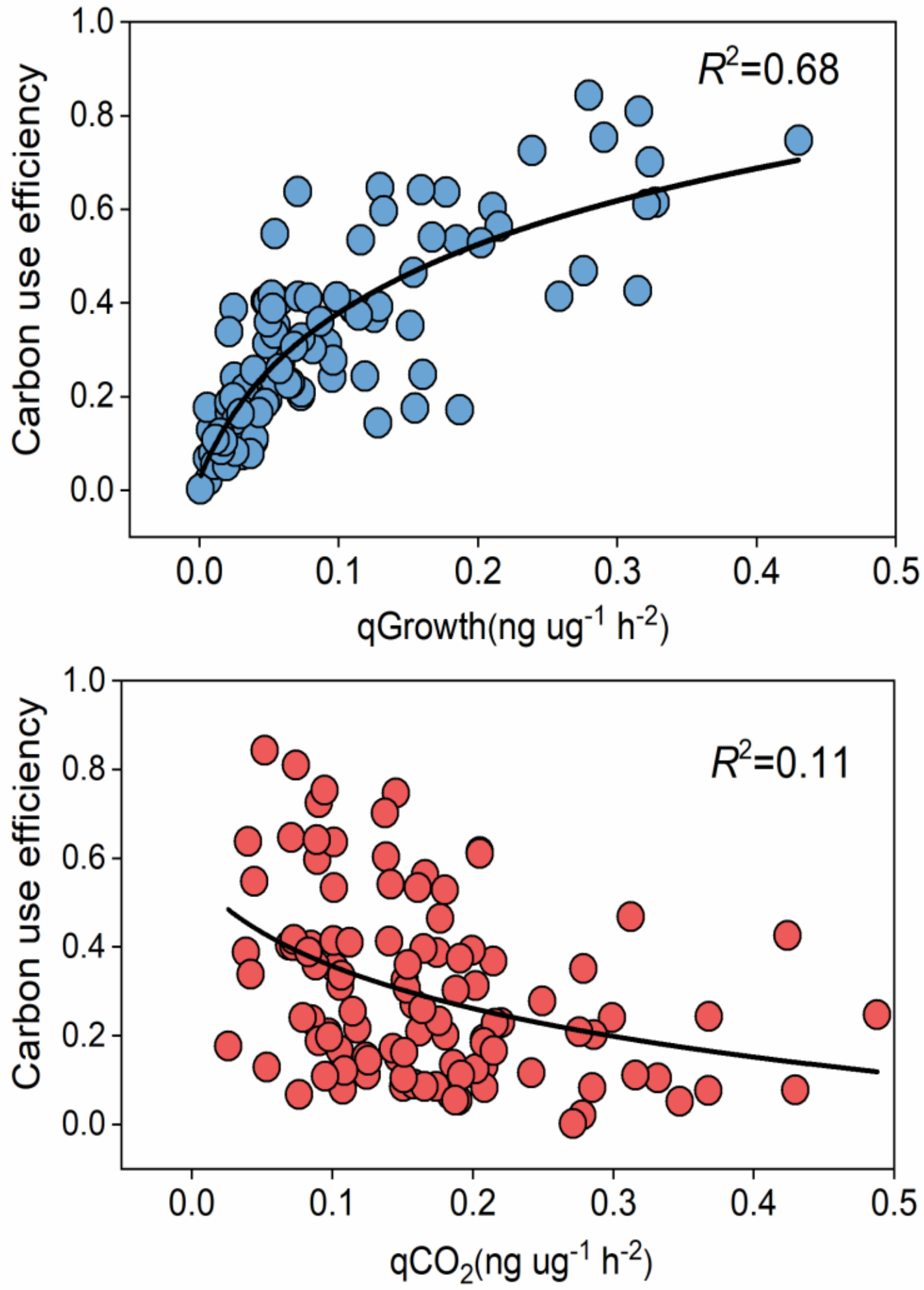

Supplementary Fig. 6 Relationships between qGrowth, qCO_2 and carbon use efficiency. qGrowth, microbial biomass-based growth rates; qCO_2, metabolic quotients.

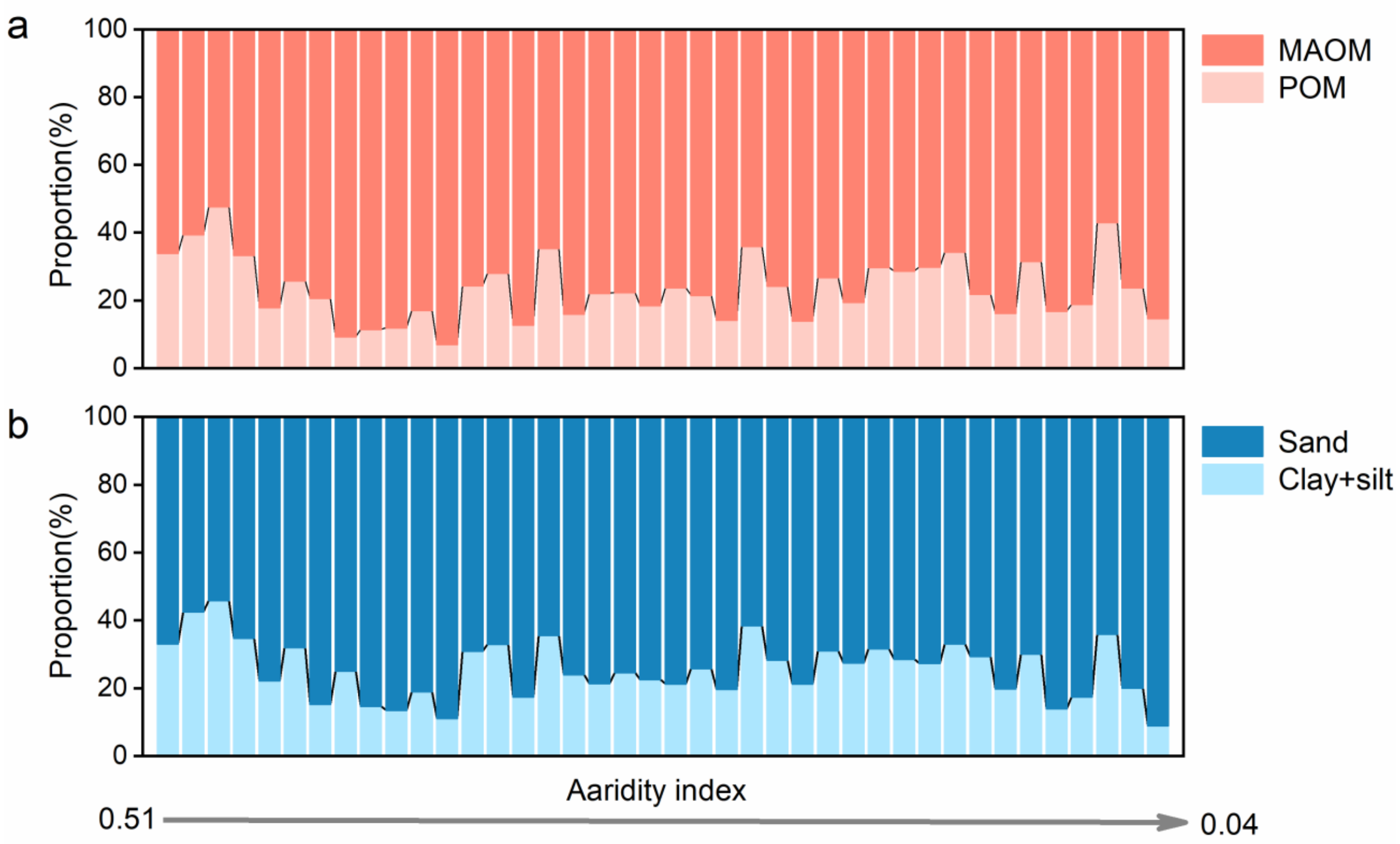

Supplementary Fig. 7 Relative weight ratio of soil density fractionation (a) and soil texture (b)

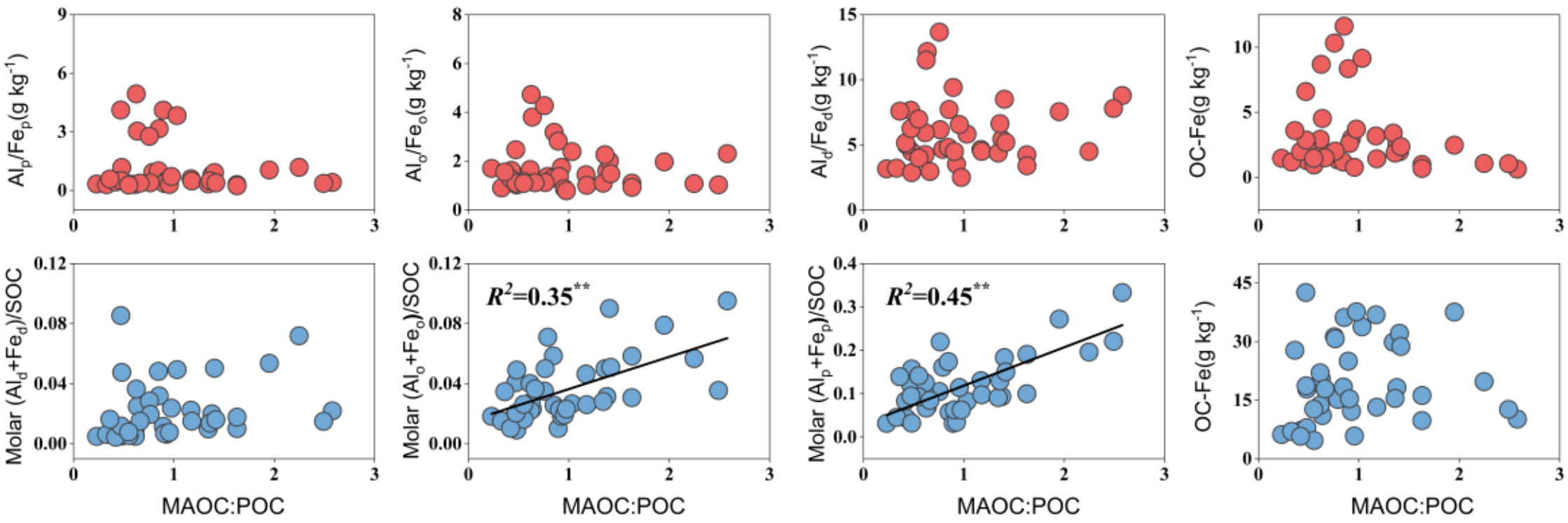

Supplementary Fig. 8 Correlations between iron-aluminum oxides and the ratio of MAOC to POC.

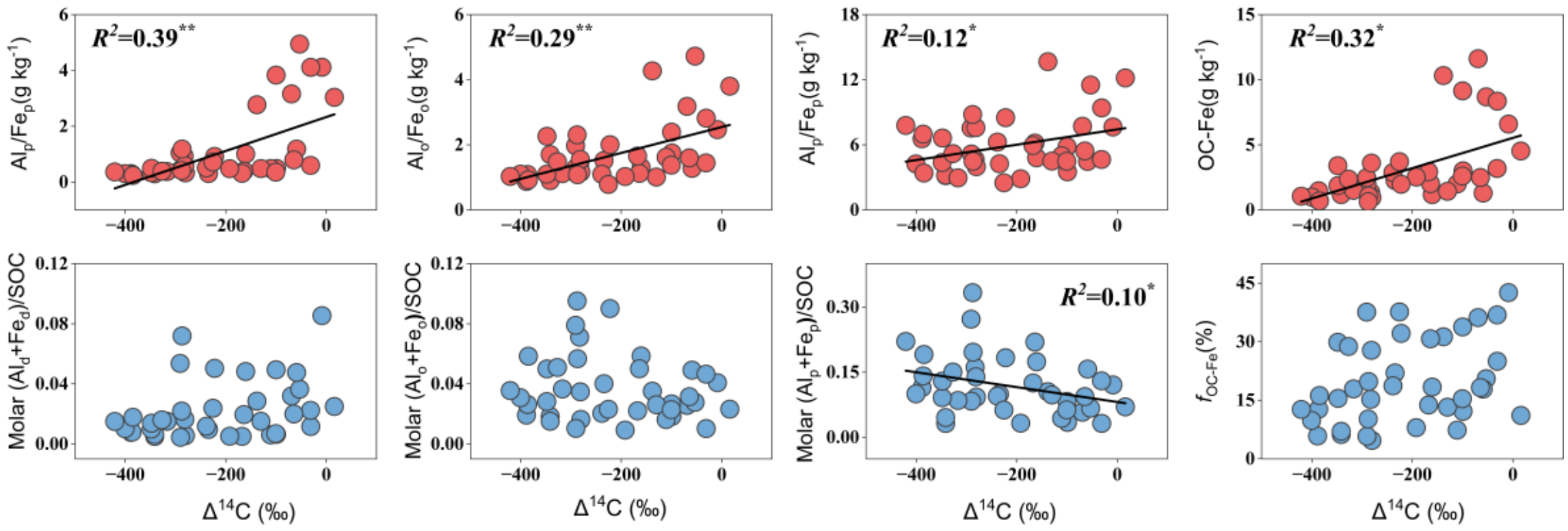

Supplementary Fig. 9 Correlations between iron-aluminum oxides and soil carbon persistence ($\Delta^{14}C$).

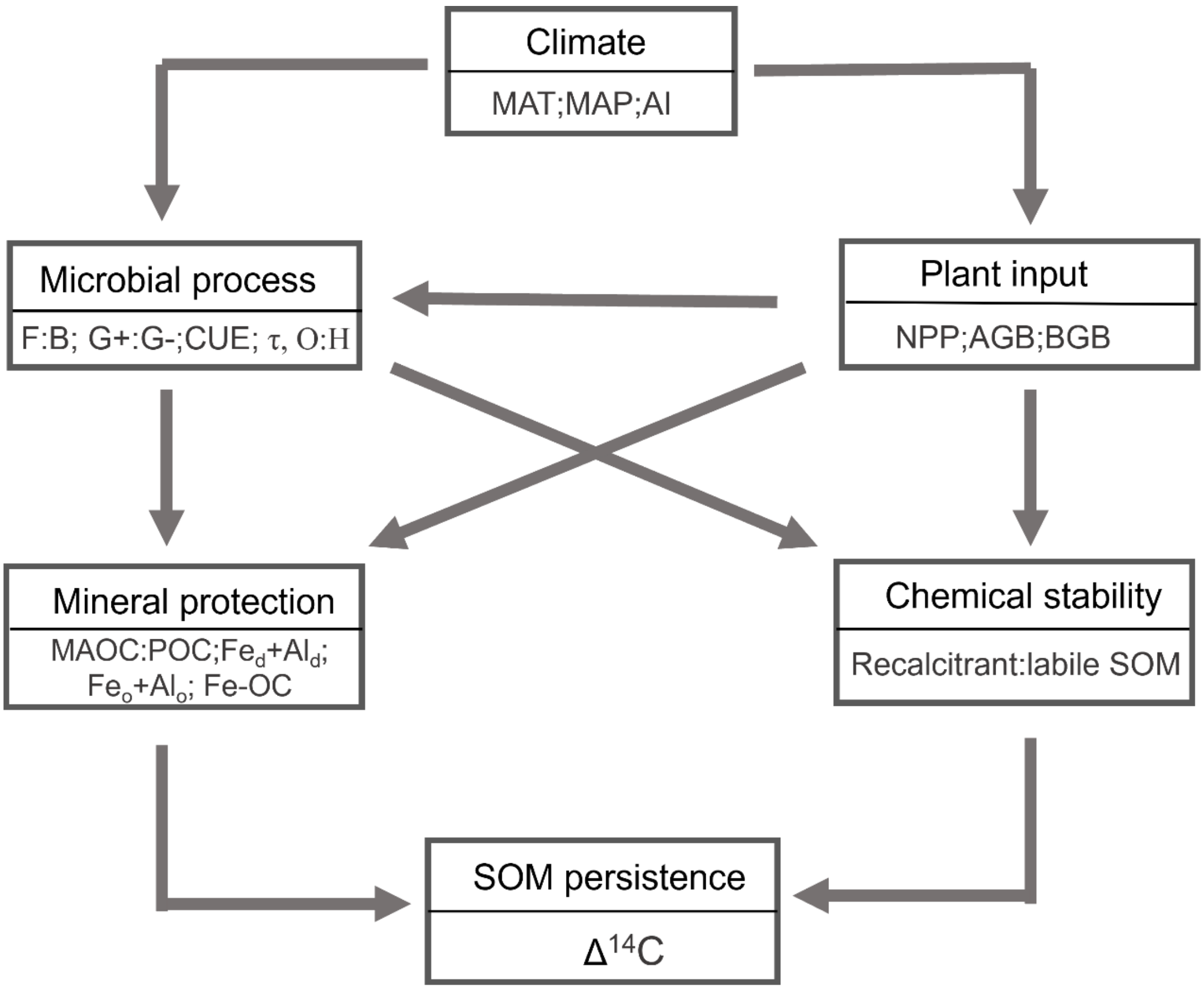

Supplementary Fig. 10 A priori model for piecewise structural equation modeling (SEM) analysis of soil organic matter persistence along the grassland transect. Arrows represent the directional influence of one variable upon another. MAT, mean annual temperature; MAP, mean annual precipitation; AI, aridity index; NPP, net primary production; AGB, aboveground biomass; BGB, belowground biomass; F:B, the ratio of fungi to bacteria; G+: G-, the ratio of Gram-positive bacteria to Gram-negative bacteria; CUE, carbon use efficiency; τ, microbial biomass turnover rate; MAOC:POC, ratio of mineral-associated carbon to particulate organic carbon; O:H, the ratio of oxidase to hydrolase; Al_d+Fe_d, reactive free oxides; Fe_o+Al_o, amorphous oxide; OC-Fe, iron-associated organic carbon.

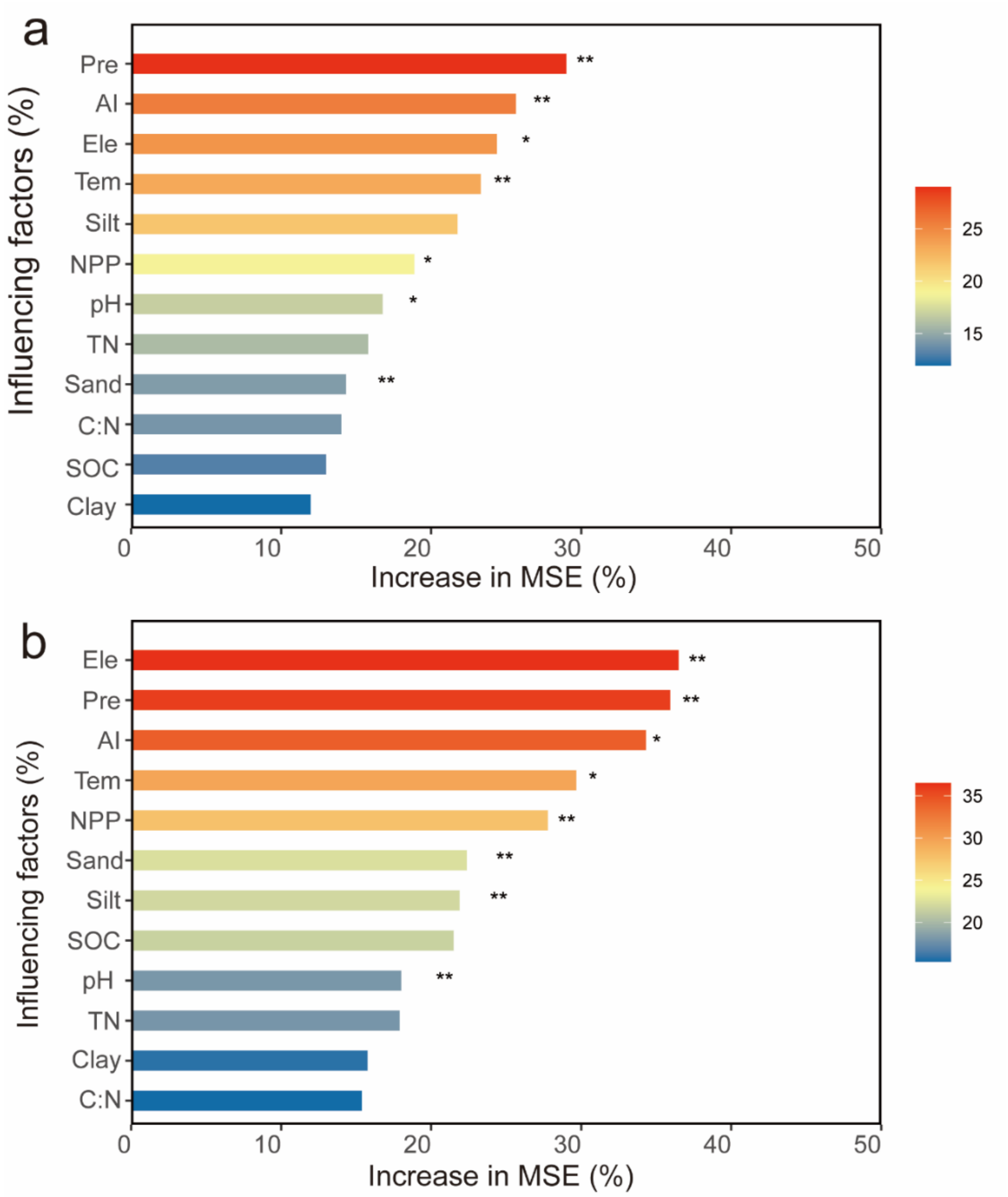

Supplementary Fig. 11 Relative importance of environmental variables in influencing MAOC/POC ratio (**a**) and $\Delta^{14}C$ (**b**) revealed by random forest model. The data of MAOC/POC ratio and $\Delta^{14}C$ were extracted from the global soil fractionation and radiocarbon dataset and environmental variables were extracted from the relevant global dataset (see method). Pre, mean annual precipitation; Tem, mean annual temperature; AI, aridity index; Ele, elevation; NPP, net primary production; SOC, soil organic carbon; TN, soil total nitrogen; C:N, the ratio of total carbon to nitrogen in soil; * indicates significant difference at the 0.05 level; ** indicates significant difference at the 0.01 level.

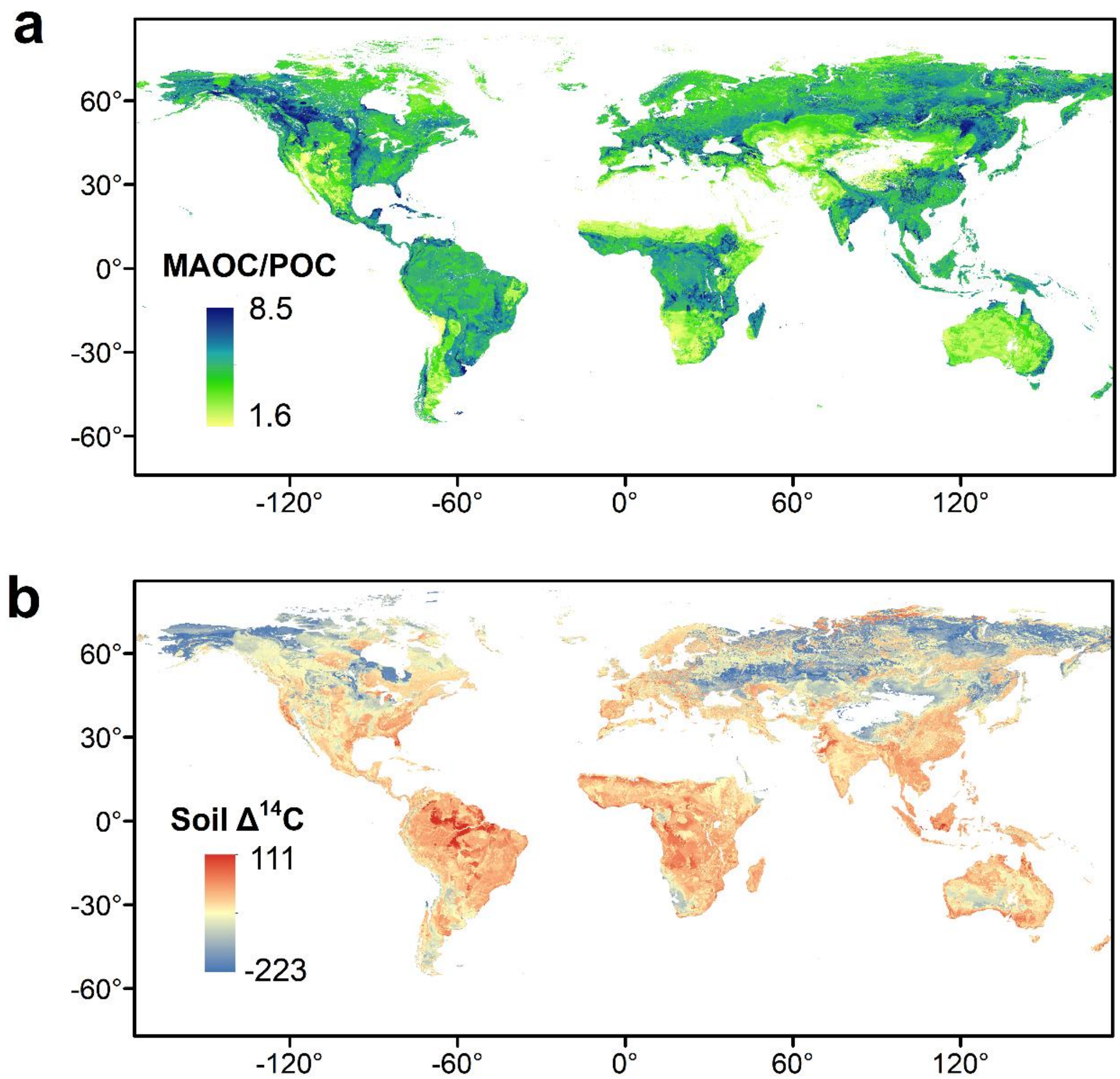

Supplementary Fig. 12 Global predicted spatial pattern of surface soil organic matter persistence, indicated by the MAOC to POC ratio (**a**) and soil $\Delta^{14}C$ values (**b**) across the globe at the resolution of 0.0083°. MAOC, mineral-associated organic carbon; POC, particulate organic carbon.

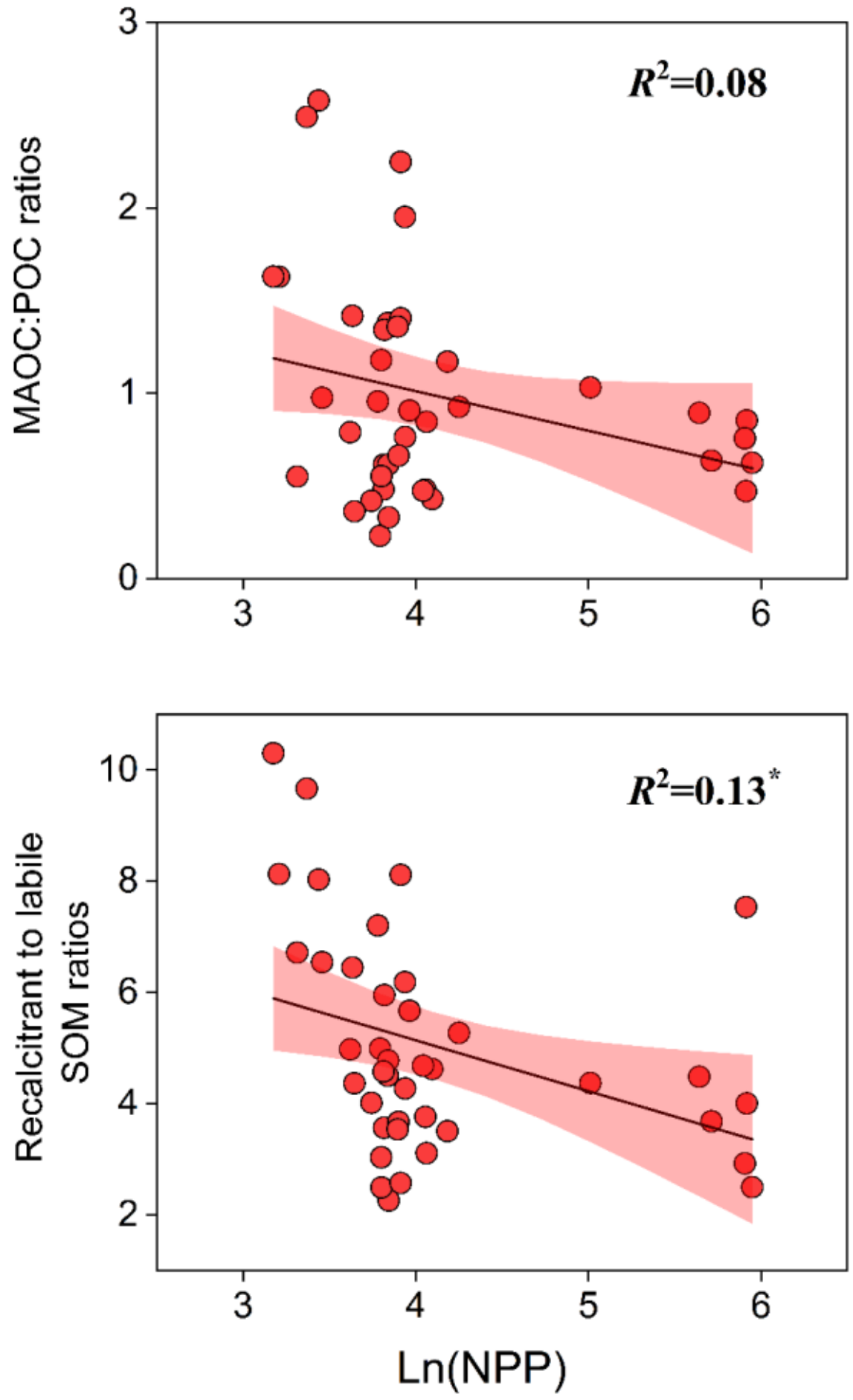

Supplementary Fig. 13 Relationships of MAOC:POC ratio and recalcitrant to labile SOM ratio with NPP.

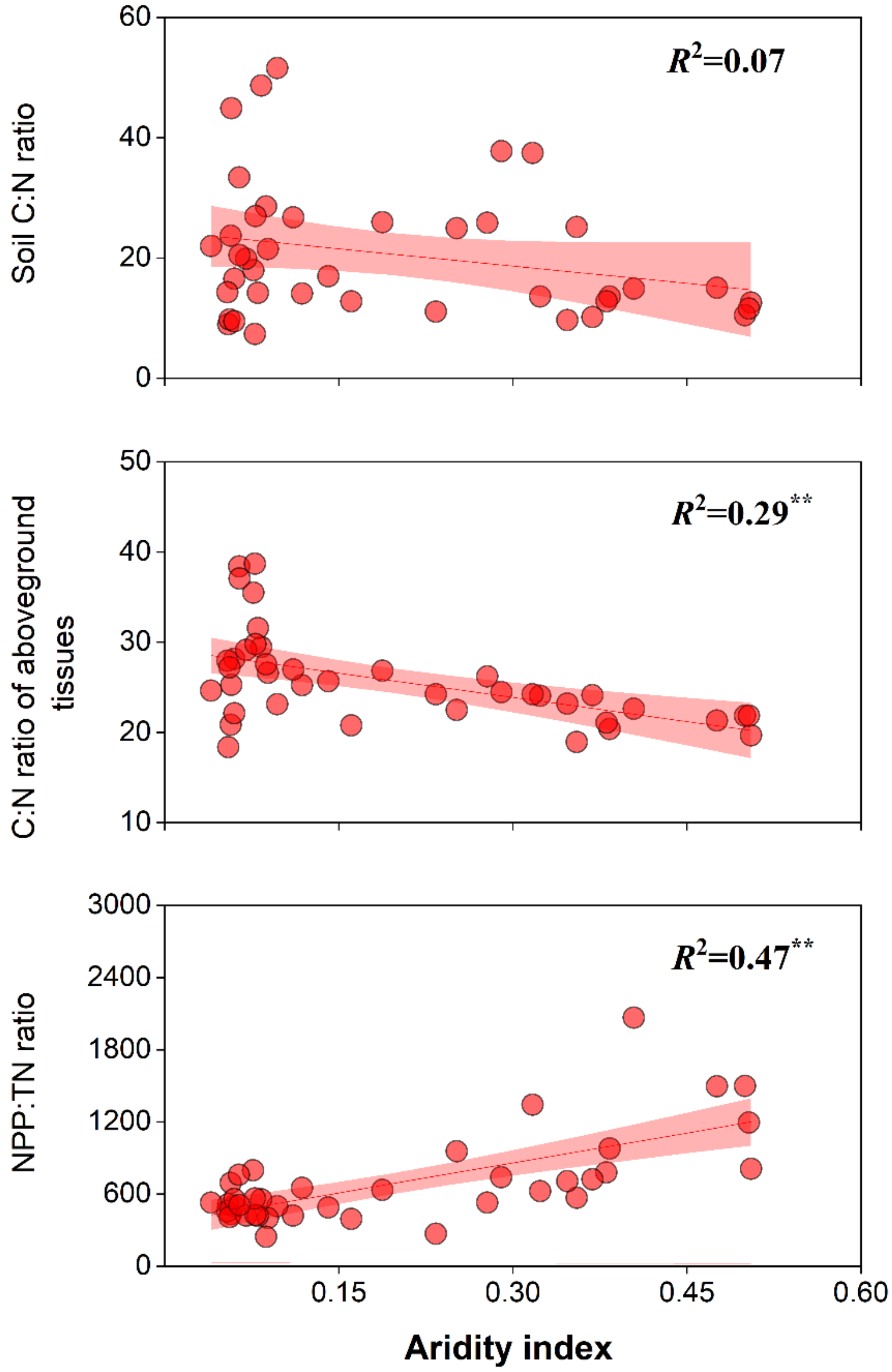

Supplementary Fig. 14 Stoichiometry of plant and soil along the transect.

Reference

1. Q. Zheng, Y. Hu, S. Zhang, L. Noll, T. Böckle, A. Richter, W. Wanek, Growth explains microbial carbon use efficiency across soils differing in land use and geology. *Soil Biol. Biochem.* **128**, 45-55 (2019).
2. B. Engelking, H. Flessa, R. G. Joergensen, Shifts in amino sugar and ergosterol contents after addition of sucrose and cellulose to soil. *Soil Biol. Biochem.* **39**, 2111-2118 (2007).
