# Supplementary material for: Declines in organic matter persistence with increased soil carbon
Source: Nat Commun. 2026 Jul 7;17:8521. doi: 10.1038/s41467-026-75185-4 (PMC13482611; doi:10.1038/s41467-026-75185-4)
Supplement: Supplementary file 2 [file 41467_2026_75185_MOESM2_ESM.pdf]

nature portfolio

Corresponding author(s): Yangjian Zhang

Last updated by author(s): 2026/04/03

Reporting Summary

Nature Portfolio wishes to improve the reproducibility of the work that we publish. This form provides structure for consistency and transparency in reporting. For further information on Nature Portfolio policies, see our Editorial Policies and the Editorial Policy Checklist.

Statistics

For all statistical analyses, confirm that the following items are present in the figure legend, table legend, main text, or Methods section.

n/a	Confirmed	
☐	☒	The exact sample size (*n*) for each experimental group/condition, given as a discrete number and unit of measurement
☐	☒	A statement on whether measurements were taken from distinct samples or whether the same sample was measured repeatedly
☐	☒	The statistical test(s) used AND whether they are one- or two-sided<br>*Only common tests should be described solely by name; describe more complex techniques in the Methods section.*
☒	☐	A description of all covariates tested
☒	☐	A description of any assumptions or corrections, such as tests of normality and adjustment for multiple comparisons
☐	☒	A full description of the statistical parameters including central tendency (e.g. means) or other basic estimates (e.g. regression coefficient) AND variation (e.g. standard deviation) or associated estimates of uncertainty (e.g. confidence intervals)
☐	☒	For null hypothesis testing, the test statistic (e.g. *F*, *t*, *r*) with confidence intervals, effect sizes, degrees of freedom and *P* value noted<br>*Give P values as exact values whenever suitable.*
☒	☐	For Bayesian analysis, information on the choice of priors and Markov chain Monte Carlo settings
☒	☐	For hierarchical and complex designs, identification of the appropriate level for tests and full reporting of outcomes
☐	☒	Estimates of effect sizes (e.g. Cohen's *d*, Pearson's *r*), indicating how they were calculated

Our web collection on statistics for biologists contains articles on many of the points above.

Software and code

Policy information about availability of computer code

Data collection: No software was used to collect data, as no new data were generated for this study

Data analysis: Data analysis for this study was performed using a combination of open-source and commercial software.
Statistical analyses, including linear mixed models, Mantel tests, and Random Forest analysis, were conducted using R version 4.2.2.Structural equation modeling (SEM) was conducted using a piecewise framework implemented in the R package piecewiseSEM.

For manuscripts utilizing custom algorithms or software that are central to the research but not yet described in published literature, software must be made available to editors and reviewers. We strongly encourage code deposition in a community repository (e.g. GitHub). See the Nature Portfolio guidelines for submitting code & software for further information.

Data

Policy information about availability of data

All manuscripts must include a data availability statement. This statement should provide the following information, where applicable:

- Accession codes, unique identifiers, or web links for publicly available datasets
- A description of any restrictions on data availability
- For clinical datasets or third party data, please ensure that the statement adheres to our policy

The datasets supporting the findings are available in the figshare repository (https://doi.org/10.6084/m9.figshare.28636262)

Research involving human participants, their data, or biological material

Policy information about studies with human participants or human data. See also policy information about sex, gender (identity/presentation), and sexual orientation and race, ethnicity and racism.

Reporting on sex and gender	This study does not involve research on human participants, animals, or biological materials, and therefore sex and gender considerations are not applicable.
Reporting on race, ethnicity, or other socially relevant groupings	This study does not involve human participants or related data, and therefore reporting on race, ethnicity, or other socially relevant groupings is not applicable.
Population characteristics	This study does not involve human participants, and therefore no population characteristics are applicable.
Recruitment	This study did not involve any human participants, and therefore no recruitment process was applicable
Ethics oversight	Ethical approval was not required for this study as it did not involve human participants, animal subjects, or the collection of specimens.

Note that full information on the approval of the study protocol must also be provided in the manuscript.

Field-specific reporting

Please select the one below that is the best fit for your research. If you are not sure, read the appropriate sections before making your selection.

☐ Life sciences ☐ Behavioural & social sciences ☒ Ecological, evolutionary & environmental sciences

For a reference copy of the document with all sections, see nature.com/documents/nr-reporting-summary-flat.pdf

Ecological, evolutionary & environmental sciences study design

All studies must disclose on these points even when the disclosure is negative.

Study description	Focused on soil samples from a 1500-km grassland-dominated transect on the Tibetan Plateau, with gradients in mean annual precipitation (50–650 mm), mean annual temperature (−3.6–2.27 °C), and aridity, to investigate microbial pathways in SOM formation and stabilization. Included 40 sampling sites, each with 5 replicate 10 m × 10 m plots (3 1 m × 1 m quadrats per plot). Topsoil (0–10 cm) was composited, sieved (2 mm), and 3 replicates per site were randomly chosen for analysis. Above/belowground biomass and NPP were also measured.
Research sample	Soil samples from a grassland transect on the Tibetan Plateau. Chosen to capture wide environmental gradients for studying microbial impacts on SOM, representing grassland soils from cold-arid to temperate climates, sourced from 40 sites with surface litter manually removed
Sampling strategy	40 sites were set in July 2020 (40–50 km apart). Each site had 5 plots (10 m × 10 m, >20 m apart) with 3 quadrats (1 m × 1 m) each. Sample sizes were selected to cover broad gradients and ensure representativeness.
Data collection	Researchers removed surface litter, collected topsoil (0–10 cm) via a 70-mm auger from each quadrat, composited samples, sieved (2 mm), and randomly picked 3 replicates per site for lab analysis. Root samples (from soil cores) were cleaned/dried for belowground biomass; aboveground biomass was measured by clipping
Timing and spatial scale	Data collected in July 2020, spanning a 1500-km grassland transect on the Tibetan Plateau. Sites were 40–50 km apart, plots 10 m × 10 m, quadrats 1 m × 1 m, soil depth 0–10 cm. Chosen to cover environmental gradients using a space-for-time approach
Data exclusions	No data were excluded.
Reproducibility	A reproducible sampling design, involving systematic collection of soil and plant replicates at 40 sites along a 1500-km grassland transect, was implemented to ensure robust analysis.
Randomization	Three replicates per site were randomly selected for lab analysis. As an observational study, group allocation wasn't applicable.
Blinding	Blinding was not applicable to our study as all data collected were objective physical and chemical measurements of environmental samples.

Did the study involve field work? ☒ Yes ☐ No

Field work, collection and transport

Field conditions	The field work was conducted in a grassland-dominated transect on the Tibetan Plateau. The transect has a gradient of mean annual precipitation (50–650 mm) and mean annual temperature (−3.6–2.27 °C) from east to west
Location	Sampling was performed in a 1500-km-long grassland transect on the Tibetan Plateau, covering grasslands from cold/arid to temperate climates. Specific latitude and longitude details were not provided.
Access & import/export	In compliance with local and national regulations, all soil and plant samples for this study were collected with the necessary permissions. Sampling was conducted across a 1500-km grassland transect on the Tibetan Plateau in July 2020.
Disturbance	To minimize disturbance, surface litter was manually removed before sampling. Soil was collected using a soil auger, and plots were spaced >20 m apart, reducing impacts on the grassland ecosystem.

Reporting for specific materials, systems and methods

We require information from authors about some types of materials, experimental systems and methods used in many studies. Here, indicate whether each material, system or method listed is relevant to your study. If you are not sure if a list item applies to your research, read the appropriate section before selecting a response.

Materials & experimental systems

n/a	Involved in the study
☒	☐ Antibodies
☒	☐ Eukaryotic cell lines
☒	☐ Palaeontology and archaeology
☒	☐ Animals and other organisms
☒	☐ Clinical data
☒	☐ Dual use research of concern
☒	☐ Plants

Methods

n/a	Involved in the study
☒	☐ ChIP-seq
☒	☐ Flow cytometry
☒	☐ MRI-based neuroimaging

Plants

Seed stocks	Plant materials, including aboveground biomass and root samples, were collected in July 2020 from a 1500-km grassland-dominated transect encompassing 40 sampling sites on the Tibetan Plateau.
Novel plant genotypes	This study did not involve the production of novel plant genotypes via transgenic approaches, gene editing, chemical/radiation-based mutagenesis, or hybridization.
Authentication	No authentication procedures were performed for seed stocks or novel genotype.
