# Supplementary material for: Declines in organic matter persistence with increased soil carbon
Source: Nat Commun. 2026 Jul 7;17:8521. doi: 10.1038/s41467-026-75185-4 (PMC13482611; doi:10.1038/s41467-026-75185-4)
Supplement: Supplementary file 3 [file 41467_2026_75185_MOESM3_ESM.pdf]

nature portfolio

Peer Review File

Declines in organic matter persistence with increased soil carbon

Corresponding Author: Dr Yangjian Zhang

This file contains all reviewer reports in order by version, followed by all author rebuttals in order by version.

Version 0:

Reviewer comments:

Reviewer #1

(Remarks to the Author)
Comments to Authors
This study investigates the relationship between microbial carbon use efficiency (CUE) and soil organic matter (SOM) persistence by integrating soil radiocarbon, SOM physicochemical characterization, and CUE measurements across surface soils along an environmental gradient, supplemented by global-scale datasets. The authors report that as soil organic carbon (SOC) content increases, its persistence—inferred from radiocarbon age, MAOC:POC and recalcitrant : labile SOM ratios—declines. Using Mantel tests and structural equation modeling, they attribute this pattern to enhanced microbial utilization and recycling under higher nutrient demands in SOC-rich soils. This study reveals a decoupled pattern between CUE and microbial necromass carbon accumulation in soils, challenging the prevailing view that higher CUE leads to greater preservation of microbial necromass carbon towards higher SOC storage. The topic is interesting. However, I have some comments.
First of all, the study attributes the negative correlation between CUE and microbial necromass carbon (or SOM persistence, characterized by its physicochemical properties), primarily to microbial properties (e.g., microbial community composition, enzyme activities, and CUE). However, microbial necromass carbon accumulation represents a balance between microbial necromass carbon production and preservation processes. While clay and silt contents showed no significant correlation with MAOC:POC or recalcitrant : labile SOM ratios in this study, this does not necessarily imply that mineral protection mechanisms (e.g., association with Fe oxides) play a negligible role in microbial necromass carbon accumulation. To strengthen the conclusions, I recommend either: (1) direct experimental validation—such as quantifying reactive Fe/Al oxides (e.g., via dithionite-citrate-bicarbonate extraction or oxalate extraction for short-range-order minerals) to assess their association with SOM persistence; or (2) explicit discussion—acknowledging potential mineral protection pathways (e.g., Fe-organic matter association and ligand exchange) that may operate independently of bulk clay/silt content, particularly in systems where microbial and mineral controls interact.
Second, the current operational definition of chemical stability, that classifying recalcitrant SOM as microbial-derived carbon, while labeling all plant-derived carbon as labile, appears problematic, because this dichotomy contradicts established understanding that certain plant-derived biomolecules (e.g., lignin and long-chain alkyl compounds) exhibit well-documented recalcitrance in soil systems. The very fact that specialized lignin-modifying oxidases are required for their decomposition - as noted in your study - inherently validates their recalcitrant nature.
Third, radiocarbon age represents a fundamental metric for assessing SOM persistence. Its exclusion from key analyses (e.g., structural equation modeling) raises some questions. Specifically: (1) How does radiocarbon age relate to MAOC:POC and recalcitrant : labile SOM ratios? (2) What is its association with microbial CUE? Clarifying these relationships would provide critical insights into whether MAOM:POC and recalcitrant : labile SOM ratios accurately reflect SOM persistence.

I also have pointed out some mistakes below.
Detailed comments:
1. Replace “microbially-derived” by “microbial-derived” throughout the manuscript.
2. Line 58: The parenthetical phrase "(hereafter 'microbial necromass')" should be deleted, as microbial-derived SOM or carbon is a more commonly used term in this context.
3. Line 89, Please clarify the conceptual relationship between "organic C" (a general term) and "necromass-derived C" (a specific fraction) in this context.
4. Line 153, delete “aridity index”.
5. Line 169-173, “R=” should be “r=”.

6. Line 262, delete “environments”.
7. In Figures, please ensure consistent notation between images and captions. For example, use either "MAOC to POC ratios" or "MAOC:POC", and "O/H" or "O:H".

Reviewer #2

(Remarks to the Author)
This manuscript titled “Declines in organic matter persistence with increased soil carbon regulated by microbes” provides valuable datasets about Δ14C and SOM fractions at regional and global scale. The authors explore the drivers of SOM persistence and conclude that SOM persistence declines with SOC. They also highlight the importance of microbial traits in affecting SOC stabilization. I appreciate the authors’ efforts in determining important microbial traits such as CUE and necromass, but have some concerns about writing, data interpretation, and statistical analysis.
1 My first concern is the definition and representation of SOM persistence in this study. Δ14C is an integrative parameter of carbon persistence and is indeed measured in the MS. But in the following analyses (regression, mantel test, random forest, and SEM), physical protection and chemical recalcitrance, rather than Δ14C, are explained by the biotic and abiotic factors. What’s the relationship between Δ14C and the predictors? Also, as mentioned by the authors in the Introduction, mineral protection and chemical recalcitrance play important roles in affecting SOM persistence (lines 81-83). From these statements, they may not be equal to SOM persistence. Do authors think these two parameters could represent SOM persistence?
2 The authors stress the importance of microbial properties particularly microbial CUE in affecting SOM persistence. But I’m not sure the results support the conclusions, as other factors like soil properties are even more important as shown by the results (Figs. 2-3). Perhaps more evidence is needed to draw the present conclusion. Otherwise, I think the authors over-interpret microbial effects.
3 As an important parameter of the manuscript, the calculation of ratio of recalcitrant to labile SOM need to be justified. The broad classification of plant-derived components into labile and microbial-derived into recalcitrant seems unreasonable and I think is not supported by the reference cited by the authors.
4 While the authors try to use the global-scale data to confirm the findings from the regional scale, only the relationships of MAOC/POC and Δ14C with SOC are presented. Could this support the main conclusion of this study? Do the authors mainly focus on the effects of SOC or microbial effects?
5 Some statements in the Introduction and Discussion are not closely related to the findings or not within the scope of this study. For example, the authors mention necromass production and stabilization, formation pathways of persistent soil C, and the stabilization and destabilization of MAOM for many times, which are not determined in this study. The authors need to better organize the contents to closely match the findings.

Specific comments:
-The title is not very clear. What does “regulated by microbes” mean? Regulate what?
-The results described in the Abstract (Soil microbial biomass, CUE and turnover rate positively correlate with SOC, but do not enhance preservation of microbially-derived carbon in soils, suggesting decoupling of microbial necromass production from stabilization) are not the main results shown in the MS.
-Line 60, in this study, mineral protection is not considered as a predictor of SOM persistence parallel with microbial traits but seems as a proxy of SOM persistence.
-Lines 102-108, the aim and hypotheses mentioned here are not closely related to the results of the study.
-Lines 152-153, CUE aridity index?
-Lines 204-205, it should be a positive relationship in Fig. 5c.
-Lines 216-218, the negative relationship between necromass and CUE is because that the authors use the necromass per unit SOC. What about the absolute amount of necromass? I guess it should be a positive relationship, and I’m not sure the results challenge the widely accepted theory.
-Lines 228-238, this paragraph reads unclear.
-Lines 309-318, like Abstract, I think the summary does not include the main results and is not within the scope of this study. How could the authors get the conclusion: “this study reveals a decoupled pattern between CUE and microbial necromass accumulation in soils, challenging the prevailing view that higher CUE leads to greater preservation of microbially-derived C towards higher SOC storage”?
-Line 346, here the authors describe the method for total C. What about soil organic carbon?
-Lines 361-362, provide more details. What’s the unit for POC and MAOC?
-Lines 364-365, are the amino sugars in bulk soil measured or only for the two fractions?
-Lines 410-414, are Gram-negative bacteria and Gram-positive bacteria included in bacteria when calculating F/B? The name of the biomarkers should be written accurately.
-Lines 417-420, what’s the soil water content during preincubation and CUE measurement?
-Line 482, what’s the fixed effect and random effect in the linear mixed model?
Supplementary materials:
-The text should be cited in the MS.
-Fig. S6, in a priori model, the authors do not consider the effect of climate on plant, but the effect exists in the final model. Also, I think some effects are missed in a priori model such as soil properties to microbial traits and microbial traits to enzyme activity.

Version 1:

Reviewer comments:

Reviewer #1

(Remarks to the Author)
I have reviewed the revised manuscript entitled "Declines in Organic Matter Persistence with Increased Soil Carbon" by Zhao et al. As one of the original reviewers, I would like to commend the authors for their comprehensive and thoughtful responses to the previous comments. The manuscript has been significantly improved, and I am satisfied with the revisions. Before final publication, I would like to suggest the following minor technical corrections to further enhance clarity and precision:
1) Line 90: Consider adding "by" before "enhanced," as the current phrasing appears grammatically incomplete. The sentence should be reviewed and revised for clarity.
2) Line 143-144, The abbreviations Ald, Fed, Alo, and Feo are used here for the first time. To ensure readability for a broad audience, please provide their full names upon initial use.
3) Line 148-149, Although "recalcitrant" and "labile" are defined in the Method section, it would be beneficial to explicitly specify these terms in the main text as well. Please clearly indicate which compound classes (e.g., lignin, aliphatic biopolymers vs. sugars, proteins) are classified as recalcitrant and labile in the context of this study, supported by relevant references.

Reviewer #2

(Remarks to the Author)
I acknowledge the authors' efforts and improvements during the last round of revision, but think they have not fully addressed some of the issues raised earlier.
1 The authors state in the revised MS that their findings challenge the paradigm that C-rich soils are inherently more stable. I believe the traditional paradigm did not establish such relationship between carbon content and stability, and the authors' statement would be misleading to readers.
2 Although the authors have added new analyses for Δ14C, how they use Δ14C with MAOM and molecular-level recalcitrance to represent SOM persistence is still unclear. In the Introduction, the authors use a long paragraph to introduce MAOM, which gives impression that this manuscript mainly focuses on MAOM. However, current knowledge about Δ14C is not included in the Introduction. In lines 124-138, the authors refer MAOM and molecular-level recalcitrance as potential mechanisms of SOM persistence, but in Fig. 2b, they use Δ14C as predictor of physical and chemical composition. In addition, MAOM was not included in the SEM. These points are confused and the authors need to clearly organize these content and results.
3 Both random forest and SEM reveal the importance of NPP in affecting SOM persistence, but this effect is not discussed in current MS. Instead, the authors used long paragraphs to discuss microbial pathways driving MAOM recycling, which is not closely related to the main topic of the MS and lacks direct experimental evidence. Particularly, the statements regarding nutrient limitation need to be supported by direct results.
4 There are still errors in the revised MS. For example, line 217, it should be increase but not decrease. The arrow between chemical stability and SOM persistence was missed in a priori model in Fig. S9, but shown in the final SEM. These errors should be avoided in high quality papers in Nature Communications.

Version 2:

Reviewer comments:

Reviewer #1

(Remarks to the Author)
I have re-reviewed the revised manuscript by Zhao et al. The authors have responded thoughtfully to all prior comments, and the manuscript is greatly improved. I am happy to recommend it for publication.

Reviewer #2

(Remarks to the Author)
The authors have done a good job addressing the concerns raised in the previous round. I have the following minor comments on the revised manuscript.
1 Line 120: Does this sentence imply that soil pH is higher in sites with higher NPP? Given that soil pH is typically negatively associated with SOC, this description is somewhat surprising
2 Lines 137-138: I suggest mentioning here that microbial-derived carbon was determined for POM and MAOM fractions separately to make this information clearer
3 Lines 142-146: as mentioned by the authors, the contents of Ald, Fed and OC-Fe exhibited opposite patterns compared to the molar ratios. Which metrics are ultimately used to represent the capacity of mineral protection?
4 Lines 173-176: the sentences are incomplete

Version 3:

Reviewer comments:

Reviewer #2

(Remarks to the Author)
The authors have adequately addressed the concerns raised in the previous round of review, and I have no further comments.

Open Access This Peer Review File is licensed under a Creative Commons Attribution 4.0 International License, which permits use, sharing, adaptation, distribution and reproduction in any medium or format, as long as you give appropriate credit to the original author(s) and the source, provide a link to the Creative Commons license, and indicate if changes were made.
In cases where reviewers are anonymous, credit should be given to 'Anonymous Referee' and the source.
The images or other third party material in this Peer Review File are included in the article’s Creative Commons license, unless indicated otherwise in a credit line to the material. If material is not included in the article’s Creative Commons license and your intended use is not permitted by statutory regulation or exceeds the permitted use, you will need to obtain permission directly from the copyright holder.
To view a copy of this license, visit https://creativecommons.org/licenses/by/4.0/

Reviewer #1 (Remarks to the Author):

Comments to Authors

This study investigates the relationship between microbial carbon use efficiency (CUE) and soil organic matter (SOM) persistence by integrating soil radiocarbon, SOM physicochemical characterization, and CUE measurements across surface soils along an environmental gradient, supplemented by global-scale datasets. The authors report that as soil organic carbon (SOC) content increases, its persistence—inferred from radiocarbon age, MAOC:POC and recalcitrant: labile SOM ratios—declines. Using Mantel tests and structural equation modeling, they attribute this pattern to enhanced microbial utilization and recycling under higher nutrient demands in SOC-rich soils. This study reveals a decoupled pattern between CUE and microbial necromass carbon accumulation in soils, challenging the prevailing view that higher CUE leads to greater preservation of microbial necromass carbon towards higher SOC storage. The topic is interesting. However, I have some comments.

[Response]: We are grateful for the reviewer's insightful comments, which have significantly strengthened our manuscript. We have addressed all concerns, most notably by adding new experimental data on reactive Fe/Al oxides, which has been pivotal in validating and refining our conclusions.

[Comment 1] *First of all, the study attributes the negative correlation between CUE and microbial necromass carbon (or SOM persistence, characterized by its physicochemical properties), primarily to microbial properties (e.g., microbial community composition, enzyme activities, and CUE). However, microbial necromass carbon accumulation represents a balance between microbial necromass carbon production and preservation processes. While clay and silt contents showed no significant correlation with MAOC:POC or recalcitrant: labile SOM ratios in this study, this does not necessarily imply that mineral protection mechanisms (e.g., association with Fe oxides) play a negligible role in microbial necromass carbon accumulation. To strengthen the conclusions, I recommend either: (1) direct experimental validation—such as quantifying reactive Fe/Al oxides (e.g., via dithionite-citrate-bicarbonate extraction or oxalate extraction for short-range-order minerals) to assess*

their association with SOM persistence; or (2) explicit discussion—acknowledging potential mineral protection pathways (e.g., Fe-organic matter association and ligand exchange) that may operate independently of bulk clay/silt content, particularly in systems where microbial and mineral controls interact.

[Response]: We sincerely thank the reviewer for this insightful comment. Following the reviewer's recommendation, we have conducted direct experimental validation by quantifying various forms of reactive Fe/Al oxides (including dithionite-citrate-bicarbonate extractable Fe/Al (Fe_d, Al_d), acid oxalate-extractable Fe/Al (Fe_o, Al_o), and pyrophosphate-extractable Fe/Al (Fe_p, Al_p), as well as Fe-bound organic carbon (Fe-OC).

Our new results consistently demonstrate that these reactive mineral phases, particularly the amorphous and organo-complexed forms, play a significant and substantial role in the preservation of soil organic matter (Fig.2a; fig. s4-s6). Specifically, we found strong positive correlations between (Fe_d+Al_d), (Fe_o+Al_o) and key indicators of SOM persistence ($\Delta^{14}C$).

These findings have now been comprehensively integrated into the revised manuscript (see new sections in Methods, Results, and Discussion). While these results confirm the importance of mineral protection mechanisms, they also suggest that in these high-SOC environments, plant-driven nutrient demand can stimulate microbial communities to decompose otherwise stabilized carbon, potentially overriding the protective capacity of the mineral matrix.

[Comment 2] *Second, the current operational definition of chemical stability, that classifying recalcitrant SOM as microbial-derived carbon, while labeling all plant-derived carbon as labile, appears problematic, because this dichotomy contradicts established understanding that certain plant-derived biomolecules (e.g., lignin and long-chain alkyl compounds) exhibit well-documented recalcitrance in soil systems. The very fact that specialized lignin-modifying oxidases are required for their decomposition - as noted in your study - inherently validates their recalcitrant nature.*

[Response]: We sincerely thank the reviewer for this critical and insightful comment.

We agree that the operational definition of chemical stability based on a simple plant-versus-microbial origin dichotomy is problematic and oversimplified. We appreciate the reviewer for correctly pointing out that this contradicts the well-established understanding that specific plant-derived biomolecules, such as lignin and long-chain alkyl compounds, are inherently recalcitrant.

In the manuscript, the identified Py-GC/MS compounds are now functionally classified into two distinct pools based directly on their molecular structure and established biochemical persistence. **The labile SOM pool** is now explicitly defined as comprising compounds that are readily metabolized by a broad range of microorganisms via hydrolytic enzymes. This pool includes polysaccharides (marked by pyrolysis products such as furans, furfurals, and pyran derivatives) and proteins (indicated by pyrroles, pyridines, and indoles). **The recalcitrant SOM pool** is now explicitly defined as comprising compounds that require specific oxidative enzymes or physicochemical processes for degradation. This pool logically includes lignin-derived phenols, long-chain alkyl compounds, and polyaromatic hydrocarbon.

The recalcitrant to labile ratio, calculated as the sum of relative abundance of all recalcitrant pool marker to the sum of relative abundance of all labile pool markers, was applied to reflect the intrinsic chemical stability of the SOM as revealed by its molecular composition, providing a mechanistically sound indicator of its relative persistence.

Cited reference：

P. Barré, et al., The energetic and chemical signatures of persistent soil organic matter. Biogeochemistry 130, 1–12 (2016).

Q. Chen, B. Niu, Y. Hu, T. Luo, G. Zhang, Warming and increased precipitation indirectly affect the composition and turnover of labile-fraction soil organic matter by directly affecting vegetation and microorganisms. Sci. Total Environ. 714 (2020).

P. Barré, et al., Microbial and plant-derived compounds both contribute to persistent soil organic carbon in temperate soils. Biogeochemistry 140, 81–92 (2018).

Bonnefoy-Claudet, C., Kaal, J., Thevenot, M., Panettieri, M., Lévêque, J., & Mathieu, O. (2025). Soil organic matter degradability as a function of forest species, a study based on Py-GC–MS analysis. Geoderma, 459, 117374.

[Comment 3] *Third, radiocarbon age represents a fundamental metric for assessing SOM persistence. Its exclusion from key analyses (e.g., structural equation modeling) raises some questions. Specifically: (1) How does radiocarbon age relate to MAOC:POC and recalcitrant : labile SOM ratios? (2) What is its association with microbial CUE? Clarifying these relationships would provide critical insights into whether MAOM:POC and recalcitrant : labile SOM ratios accurately reflect SOM persistence.*

[Response]: We thank the reviewer for this critical insight. We fully agree that radiocarbon (^{14}C) age is a fundamental metric for assessing SOM persistence, and its relationship with the proposed operational indicators (MAOC: POC and recalcitrant: labile SOM ratio) is essential for validating our approach. In direct response to this comment, we have conducted new correlation analyses between $\Delta^{14}C$ values, SOM pool composition, and microbial carbon use efficiency (CUE). These new analyses have been added to the manuscript (Fig.1d, Fig.2a), and they provide strong support for our interpretations.

We found a significant negative correlation between soil $\Delta^{14}C$ and both the MAOC:POC ratio and the recalcitrant: labile SOM ratio (Fig.1d). This confirms that soils with a higher proportion of MAOM and 'recalcitrant' carbon were, on average, older and more persistent. This validates that these SOM physical and chemical fractions are meaningful proxies for long-term stability at the pool level. Conversely, we found a significant positive correlation between soil $\Delta^{14}C$ and microbial CUE (Fig. 2a). This indicates that systems with higher microbial CUE are associated with younger carbon at the whole-soil level. The co-existence of these two relationships is not contradictory but rather forms the cornerstone of our proposed mechanism. It demonstrates that while the mechanistic potential (via mineral association) for stabilization is intact, the net ecosystem-level outcome is a younger carbon stock due to the rapid cycle of carbon production and decomposition and high plant input in C-rich soils.

We are deeply grateful to the reviewer for this suggestion, which has led to a much more robust and compelling narrative.

I also have pointed out some mistakes below.

Detailed comments:

[Detailed comment 1] *Replace "microbially-derived" by "microbial-derived" throughout the manuscript.*

[Response]: We thank the reviewer for this helpful correction. As suggested, we have replaced "microbially-derived" with "microbial-derived" throughout the entire manuscript.

[Detailed comment 2] *Line 58: The parenthetical phrase "(hereafter 'microbial necromass')" should be deleted, as microbial-derived SOM or carbon is a more commonly used term in this context.*

[Response]: We thank the reviewer for this suggestion. We agree and have deleted the phrase "(hereafter 'microbial necromass')" and now use the more standard terms "microbial-derived SOM" or "microbial-derived carbon" throughout the manuscript.

[Detailed comment 3] *Line 89, Please clarify the conceptual relationship between "organic C" (a general term) and "necromass-derived C" (a specific fraction) in this context.*

[Response]: We sincerely thank the reviewer for this insightful comment. We agree that the relationship between these terms needed clarification in this specific context. Our intention was to highlight that the distribution of both the total soil organic C pool and a key component within it (i.e., microbial-derived C) between physical fractions influences the overall stability of soil carbon.

To address this, we have revised the sentence at line 89 (now line 67-68) as follows: " Consequently, greater allocation of organic carbon to MAOM during SOC accumulation would enhance SOM persistence."

[Detailed comment 4] *Line 153, delete “aridity index”.*

[Response]: The “aridity index” has been deleted in the sentence.

[Detailed comment 5] *Line 169-173, “R=” should be “r=”.*

[Response]: We thank the reviewer for this correction. As suggested, we have revised the statistical notation in the specified lines. We have adopted "R^2" to clearly indicate the proportion of variance explained, which is the standard and more precise metric for our regression analyses

[Detailed comment 6] *Line 262, delete “environments”.*

[Response]: Thanks for pointing out this deficiency! The “environments” has been deleted in the sentence

[Detailed comment 7] *In Figures, please ensure consistent notation between images and captions. For example, use either "MAOC to POC ratios" or "MAOC:POC", and "O/H" or "O:H".*

[Response]: We sincerely thank the reviewer for highlighting this lack of consistency, which we have now thoroughly addressed. We have conducted a comprehensive check of all figures, their captions, and the main text to ensure uniform notation.

Specifically:

We have standardized the notation for mineral-associated to particulate organic carbon ratios to "MAOC:POC" throughout the manuscript. Similarly, we have standardized the notation for the oxidases: hydrolases ratio to "O:H".

Once again, we extend our sincere gratitude to Reviewer #1 for the thorough and constructive feedback. We hope that our efforts in addressing these comments have improved the manuscript and that our revisions and responses are found to be satisfactory.

Reviewer #2 (Remarks to the Author):

This manuscript titled "Declines in organic matter persistence with increased soil carbon regulated by microbes" provides valuable datasets about Δ14C and SOM fractions at regional and global scale. The authors explore the drivers of SOM persistence and conclude that SOM persistence declines with SOC. They also highlight the importance of microbial traits in affecting SOC stabilization. I appreciate the authors' efforts in determining important microbial traits such as CUE and necromass, but have some concerns about writing, data interpretation, and statistical analysis.

[Response]: We thank the reviewer for their time and constructive comments, which have helped us improve the manuscript.

[Comment 1] *My first concern is the definition and representation of SOM persistence in this study. $\Delta^{14}C$ is an integrative parameter of carbon persistence and is indeed measured in the MS. But in the following analyses (regression, mantel test, random forest, and SEM), physical protection and chemical recalcitrance, rather than $\Delta^{14}C$, are explained by the biotic and abiotic factors. What's the relationship between $\Delta^{14}C$ and the predictors? Also, as mentioned by the authors in the Introduction, mineral protection and chemical recalcitrance play important roles in affecting SOM persistence (lines 81-83). From these statements, they may not be equal to SOM persistence. Do authors think these two parameters could represent SOM persistence?*

[Response]: We sincerely thank the reviewer for this insightful and critical comment, which helps us to clarify the core theoretical framework of our study. The reviewer is correct that $\Delta^{14}C$ is a direct, integrative measure of SOM persistence. We agree with the reviewer that physical protection and chemical recalcitrance are not equal to SOM persistence, but are instead crucial proxies for the key mechanisms that cause persistence. In direct response to this comment, we have now fully integrated $\Delta^{14}C$ into our key statistical models. The revised analysis now provides a clear and direct answer to the relationship between $\Delta^{14}C$ and the predictors.

Our new correlation analyses robustly validate that both the MAOC: POC and

recalcitrant: labile components ratios are strongly correlated with $\Delta^{14}C$ (both $p < 0.05$; Fig. 1d), with higher MAOC:POC and higher recalcitrant: labile ratios being associated with older carbon (lower $\Delta^{14}C$), confirming they are important proxies. Random Forest analysis indicated that NPP, F:B, pH, and CUE emerged as the top predictors for $\Delta^{14}C$, explaining more variance than for any other variable (Fig. 2c). This directly reveals which factors are most strongly associated with the long-term persistence of SOM in our system. We have completely reframed our SEM. The new model (Fig. 3c) now positions $\Delta^{14}C$ as the ultimate endpoint, testing the specific causal pathways through which environmental factors and microbial processes influence SOM persistence via the mediating variables of physical protection and chemical composition.

In summary, the reviewer's comment has led to a significant improvement in our work. The revised manuscript now presents a more coherent narrative: we use $\Delta^{14}C$ to define persistence, then use multivariate models to identify its key predictors, and finally use SEM to test the mechanistic pathways (physical, chemical and microbial roles) through which these predictors ultimately control SOM persistence. We are deeply grateful for this significant improvement to our work.

[Comment 2] *The authors stress the importance of microbial properties particularly microbial CUE in affecting SOM persistence. But I'm not sure the results support the conclusions, as other factors like soil properties are even more important as shown by the results (Figs. 2-3). Perhaps more evidence is needed to draw the present conclusion. Otherwise, I think the authors over-interpret microbial effects.*

[Response]: We thank the reviewer for this insightful comment, which prompted us to more carefully evaluate and articulate the relative importance of microbial properties within the broader ecosystem context. We agree that soil properties are important, as shown in our results. In response, our revised interpretation now positions microbial processes as key regulators within a framework that also includes plant input and mineral protection. In our revised manuscript, we have reframed our interpretation to present a more integrated perspective that considers plant carbon input, mineral protection, and microbial processes as interconnected drivers.

Specifically:

We acknowledge that high plant carbon input initiates a cascade of effects by intensifying nutrient demand, which subsequently shapes microbial community composition and enzyme activities. These changes in turn can regulate the efficiency of stable carbon formation. On the other hand, our new data on reactive Fe/Al oxides confirm their significant role in stabilization. The absolute content of reactive oxides was associated with younger carbon (positive correlation with $\Delta^{14}C$), indicating that high carbon inputs can saturate and override the system's overall mineral protective potential. However, the protection mechanism itself is not invalidated, as a higher molar ratio of oxides to SOC was linked to older carbon (negative correlation with $\Delta^{14}C$). Within this framework, our results consistently show that the variation in microbial properties (e.g., community composition and CUE) along the gradient exhibits a stronger statistical association with the decline in SOC persistence than the variation in mineral protective capacity alone.

Consequently, we propose that although mineral protection remains an important determinant of SOC stability, nutrients demand induced by plant input and microbial activity can emerge as a more dominant driver of its persistence in specific environments, such as the C-rich soils studied here. We have toned down our conclusions throughout the manuscript to reflect this nuanced interpretation, focusing on microbial processes as "key regulators" rather than the sole dominant driver, and we believe this now more accurately represents our findings.

[Comment 3] *As an important parameter of the manuscript, the calculation of ratio of recalcitrant to labile SOM need to be justified. The broad classification of plant-derived components into labile and microbial-derived into recalcitrant seems unreasonable and I think is not supported by the reference cited by the authors.*

[Response]: We thank the reviewer for this critical comment. We fully agree that the origin-based classification was an oversimplification. This point was also raised by Reviewer #1. Upon re-evaluating the literature on SOM molecular persistence, we have entirely redefined the operational definition with a chemistry-based classification using our Py-GC/MS data, following established literature (Barré *et al.*, 2016,2018;

Chen *et al.*, 2020; Claudet *et al.*, 2025). The recalcitrant to labile SOM ratio is now calculated based on the intrinsic molecular stability of the compounds:

(1) the labile SOM pool is now defined as comprising compounds with molecular structures that are readily metabolized, including polysaccharides and proteins.

(2) the recalcitrant pool is defined as comprising complex molecular structures that require specific oxidative enzyme, including lignin-derived phenols, long-chain alkyl compounds, and polyaromatic hydrocarbons.

We have updated the Methods, Results, and corresponding citations to reflect this more mechanistically sound approach. The Methods section now reads as:

"Based on their molecular structure and established biochemical persistence, the identified compounds were functionally classified into two distinct pools reflecting their susceptibility to microbial decomposition: (1) a labile SOM pool, comprising compounds that are readily metabolized by a broad range of soil microorganisms via hydrolytic enzymes, including polysaccharides (marked by furans, furfurals, and pyran derivatives) and proteins (indicated by pyrroles, pyridines, and indoles); and (2) a recalcitrant SOM pool, encompassing compounds that require specific oxidative enzymes for degradation, including lignin-derived phenols, long-chain alkyl compounds, and polyaromatic hydrocarbons (34, 49 – 51)."

Related references：

P. Barré, et al., The energetic and chemical signatures of persistent soil organic matter. Biogeochemistry 130, 1–12 (2016).

Q. Chen, B. Niu, Y. Hu, T. Luo, G. Zhang, Warming and increased precipitation indirectly affect the composition and turnover of labile-fraction soil organic matter by directly affecting vegetation and microorganisms. Sci. Total Environ. 714 (2020).

P. Barré, et al., Microbial and plant-derived compounds both contribute to persistent soil organic carbon in temperate soils. Biogeochemistry 140, 81–92 (2018).

Bonnefoy-Claudet, C., Kaal, J., Thevenot, M., Panettieri, M., Lévêque, J., & Mathieu, O. (2025). Soil organic matter degradability as a function of forest species, a study based on Py-GC–MS analysis. Geoderma, 459, 117374.

[Comment 4] *While the authors try to use the global-scale data to confirm the findings from the regional scale, only the relationships of MAOC/POC and Δ14C with SOC are presented. Could this support the main conclusion of this study? Do the authors mainly focus on the effects of SOC or microbial effects?*

[Response]: We thank the reviewer for this critical comment, which helps us clarify the complementary roles of our regional and global analyses. Indeed, the global datasets cannot speak directly to microbial mechanisms, a well-known limitation we acknowledge. Their purpose was to test the generality of the macroscopic ecosystem-level pattern that emerges from such mechanisms. The robust global relationships (MAOC:POC and $\Delta^{14}C$ vs. SOC) confirm that a decline in persistence with increasing SOC content is a widespread reality. This finding justifies the need for a mechanistic, process-based investigation. Our regional study was designed to uncover the microbial mechanisms behind this global pattern. We have implemented the following modifications to the main text:

(1) In the results section (when presenting the global data), we now explicitly state the purpose of this analysis: "The global-scale analyses of the SOC fraction and radiocarbon databases were employed to test the generality of the emergent relationship between SOC content and its persistence, as reflected in its physical fractionation and radiocarbon signature. While these datasets lack the resolution to elucidate the underlying microbial mechanisms, they robustly confirm that the macroscopic pattern we observed regionally is a widespread phenomenon."

(2) We have substantially rewritten the opening of the Discussion to frame our study's approach more clearly: "Our integrated regional and global analyses consistently demonstrate that SOM persistence declines with increasing SOC content. This pattern challenges the paradigm that C-rich soils are inherently more stable. The global fractionation data, showing a decreasing MAOC: POC with SOC content, indicate a systematic change in soil carbon pool composition towards faster-cycling forms. Similarly, the younger radiocarbon age (higher Δ 14C) of SOC-enriched soils directly signals a larger proportion of recently cycled carbon or a loss of old, stable carbon. Our detailed regional investigation provides a mechanistic explanation for this pattern…."

(3) we have also clarified the specific purpose of using the global datasets in the Methods section: "The purpose of this global-scale analysis was to assess the prevalence of the relationship between SOC content and its persistence, rather than to elucidate the underlying microbial mechanisms."

We hope these revisions can more accurately represent the contribution of the global data, positioning them as the foundation that justifies the need for our mechanistic regional study, rather than as direct evidence for the mechanism itself. We are grateful for the reviewer's comment, which has helped us significantly improve the clarity and logical structure of our manuscript.

[Comment 5] *Some statements in the Introduction and Discussion are not closely related to the findings or not within the scope of this study. For example, the authors mention necromass production and stabilization, formation pathways of persistent soil C, and the stabilization and destabilization of MAOM for many times, which are not determined in this study. The authors need to better organize the contents to closely match the findings.*

[Response]: We sincerely thank the reviewer for this crucial comment regarding the scope and focus of our manuscript. In response, we have undertaken a comprehensive revision of both the Introduction and Discussion to ensure all content is closely aligned with and directly supported by our specific findings.

(1) We have thoroughly reorganized the introduction to first establish the role of mineral protection before introducing microbial processes, creating a more balanced and logically flowing narrative that accurately sets the scope for our study.

(2) We now more precisely frame our hypotheses around the relationships we can test with our data: the links between SOC content, its persistence, mineral protection and microbial traits.

(3) We have removed the more speculative statements about necromass stabilization pathways that we do not directly quantify. Instead, we discuss our direct measurement: "the proportion of microbial-derived carbon in the MAOM fraction" and interpret its decline in the context of potential recycling.

We hope these revisions can sharpen the manuscript's focu and ensure that all statements are now well within the scope of our study's findings.

Specific comments:

[Specific comment 1] -*The title is not very clear. What does "regulated by microbes" mean? Regulate what?*

[Response]: We sincerely thank the reviewer for this critical comment. We agree that the original title was ambiguous and did not clearly convey the central finding of our study. The term "regulated by microbes" was intended to reflect the microbial community processes that govern the relationship between soil carbon levels and its persistence. To address this, we have revised the title to more accurately and directly state our key conclusion. Our revised title is: "Declines in organic matter persistence with increased soil carbon"

[Specific comment 2] *The results described in the Abstract (Soil microbial biomass, CUE and turnover rate positively correlate with SOC, but do not enhance preservation of microbially-derived carbon in soils, suggesting decoupling of microbial necromass production from stabilization) are not the main results shown in the MS.*

[Response]: We thank the reviewer for pointing out this discrepancy. We have rewritten the Abstract to ensure that the summarized results are the main findings presented in the manuscript. The revised Abstract now precisely states our core discovery:

"The persistence of soil organic matter (SOM) is critical for predicting terrestrial carbon-climate feedbacks. However, a mechanistic understanding of how mineral protection and microbial traits interact to control SOM persistence remains limited. Here, we analyzed soil radiocarbon, SOM physicochemical composition, and microbial CUE in surface soils along a precipitation-driven gradient in conjunction with global-scale data. As soil organic carbon (SOC) increases, its persistence based on radiocarbon age and SOM physicochemical composition declines at both the transect and global scales. While mineral protection contributes to SOC stabilization,

this pattern is primarily regulated by shifts in microbial composition and enzymatic activities in response to nutrient limitation from high plant inputs. Although positively correlated with SOC, high microbial CUE led to a lower accumulation of microbial-derived carbon in the mineral-stabilized pool, suggesting that high CUE may not guarantee long-term carbon storage. Our findings highlight the necessity to incorporate microbe-driven SOM persistence in process-based models for accurate projections of soil carbon feedback to climate change."

[Specific comment 3] *Line 60, in this study, mineral protection is not considered as a predictor of SOM persistence parallel with microbial traits but seems as a proxy of SOM persistence.*

[Response]: We thank the reviewer for this insightful comment, which helps clarify the conceptual framework of our study. We agree with the reviewer's interpretation. In the context of our study, MAOM is indeed utilized as a key indicator for long-term stabilized SOM, rather than an independent predictor variable examined in parallel with microbial traits.

To accurately reflect this, we have revised the sentence on line 60 (now line 58-60) to clarify the roles of these factors. The revised text now reads: " Therefore, a mechanistic understanding of how mineral protection and microbial traits interact to control SOM persistence is critically needed."

[Specific comment 4] *Lines 102-108, the aim and hypotheses mentioned here are not closely related to the results of the study.*

[Response]: We sincerely thank the reviewer for this critical comment. We agree that the original phrasing of the aims and hypotheses did not precisely focus on the key mechanistic finding of our study. In response, we have thoroughly revised the concluding paragraph of the Introduction (Lines 99-103) to ensure that our hypotheses are directly and unambiguously linked to the results we present thereafter.

The revised text now reads: "This study investigated the relationship between SOC

content and SOM persistence (indicated by Δ14C) along a precipitation-driven SOC gradient in grassland ecosystems of the Tibetan plateau. We hypothesized that:1) SOM persistence would decline with increasing SOC content; and 2) higher microbial CUE would enhance SOM persistence by promoting the accumulation of microbial-derived carbon in soils, particularly within the mineral-associated pool"

[Specific comment 5] *Lines 152-153, CUE aridity index?*

[Response]: We thank the reviewer for pointing out this deficiency. We have deleted the "aridity index" in the sentence.

[Specific comment 6] *Lines 204-205, it should be a positive relationship in Fig. 5c.*

[Response]: We sincerely thank the reviewer for pointing this significant error in our original description. We have corrected this error in the manuscript

[Specific comment 7] *Lines 216-218, the negative relationship between necromass and CUE is because that the authors use the necromass per unit SOC. What about the absolute amount of necromass? I guess it should be a positive relationship, and I'm not sure the results challenge the widely accepted theory.*

[Response]: We thank the reviewer for this insightful comment, which allowed us to clarify a key aspect of our findings. The reviewer is correct that the absolute amount of microbial-derived carbon (estimated from amino sugars) shows a positive correlation with SOC content (Fig. 3a), which aligns with general expectations.

However, our central argument revolves around the stabilization efficiency of this microbial necromass. We therefore focused on its proportion within the total SOC pool and more specifically, within the mineral-associated (MAOM) fraction. This relative measure reveals that in high-SOC soils, the accumulation of microbial-derived carbon does not keep pace with the overall accumulation of SOC. It is this declining proportion (or relative contribution) that correlates negatively with microbial CUE.

We have now clarified this critical distinction in the revised manuscript and included the analysis of both absolute and relative amounts in the new Figure 3. This pattern refines the simplistic view by demonstrating that a high production rate of microbial residues (implied by high CUE and correlated with higher absolute necromass) does not guarantee a proportional increase in its contribution to the long-term, mineral-stabilized pool.

We are grateful for the reviewer's question, which has enabled us to present this nuanced interpretation more clearly.

[Specific comment 8] *Lines 228-238, this paragraph reads unclear.*

[Response]: We sincerely thank the reviewer for this valuable comment. We agree that the original paragraph disrupted the flow of the discussion. In response, we have thoroughly reorganized the Discussion section to improve clarity and logical progression. Specifically, we have deleted the standalone paragraph previously located at Lines 228-238. Its core conceptual content, regarding the alignment of our findings with global patterns and the context-dependency of underlying mechanisms, has been integrated into the preceding argument (now in the revised paragraph around Lines 226-231). We hope this restructuring could enhance the clarity and coherence of the Discussion.

[Specific comment 9] *Lines 309-318, like Abstract, I think the summary does not include the main results and is not within the scope of this study. How could the authors get the conclusion: "this study reveals a decoupled pattern between CUE and microbial necromass accumulation in soils, challenging the prevailing view that higher CUE leads to greater preservation of microbially-derived C towards higher SOC storage"?*

[Response]: We thank the reviewer for this critical comment. We have entirely rewritten the summary/conclusion section to ensure it is a precise synthesis of our empirical findings and remains within the scope of our study.

The summary now reads as:

"In summary, our integrated analysis of a regional transect and global datasets provides compelling evidence that the persistence of SOM in surface soil declines as SOC content increases. This pattern is regulated by shifts in microbial community composition and extracellular enzyme activities, which are driven by increased nutrient limitation, within the broader context of mineral protection. Crucially, we demonstrate that high CUE may not enhance the preservation of microbial-derived carbon within the mineral-associated pool. This reveals that the efficiency of stabilizing microbial-derived carbon could be a more critical determinant of long-term storage than their production rate alone. Our findings highlight the necessity to incorporate this mineral-microbe interactive pathway into process-based models for accurate projections of soil carbon feedback to climate change."

[Specific comment 10] *Line 346, here the authors describe the method for total C. What about soil organic carbon?*

[Response]: We thank the reviewer for raising this important methodological point. In our study, soil organic carbon (SOC) was not measured directly but was calculated as the difference between total carbon (TC) and soil inorganic carbon (SIC), both determined by thermal decomposition using an elemental analyzer. We have now clarified this procedure in the manuscript and cited a related reference as:

"The dried soils were used for pH (H_2O), total C and N analysis (Elementar Analysensysteme, Hanau, Germany), as well as for the determination of soil organic carbon, which was calculated as the difference between total C and soil inorganic carbon. Soil

[Specific comment 11] *Lines 361-362, provide more details. What's the unit for POC and MAOC?*

[Response]: We thank the reviewer for this comment. The concentrations of both particulate organic carbon (POC) and mineral-associated organic carbon (MAOC) are expressed in the unit of g C kg^{-1} soil (grams of carbon per kilogram of dry soil). This detail has been added to the manuscript in the Methods section

[Specific comment 12] *Lines 364-365, are the amino sugars in bulk soil measured or only for the two fractions?*

[Response]: We thank the reviewer for this question. In our study, amino sugars were measured in all three categories: the bulk soil, as well as in the MAOM and POM fractions. We have revised the text in Lines 391-393 to clarify this. The modified sentence now reads:

" Microbial-derived SOC was estimated from the amount of extracted amino sugars (53) using a slightly modified procedure (54). Measurements were performed on bulk soil as well as on the isolated MAOM and POM fractions."

[Specific comment 13] *Lines 410-414, are Gram-negative bacteria and Gram-positive bacteria included in bacteria when calculating F/B? The name of the biomarkers should be written accurately.*

[Response]: We sincerely thank the reviewer for highlighting this lack of clarity and precision in our methodology. Our original lists were indeed overlapping and confusing. We have rephrased this section as:

"The PLFAs specific to fungi (18:2ω6,9c), Gram-negative bacteria (16:1ω7c, cy17:0, 18:1ω7c and cy19:0), and Gram-positive bacteria (i14:0, i15:0, a15:0, i16:0, i17:0 and a17:0) were independently quantified (Zelles, 1999). The bacterial PLFAs were calculated as the sum of Gram-negative and Gram-positive bacterial PLFAs"

[Specific comment 14] *Lines 417-420, what's the soil water content during preincubation and CUE measurement?*

[Response]: We thank the reviewer for pointing out this omission. During both the preincubation and the CUE measurement, the soil water content was adjusted to 60% of the water holding capacity (WHC). This detail has now been added to the manuscript in the respective section. Now the sentence reads as:

"For this assay, soil water content was adjusted to 60% of the water holding capacity

(WHC) and then pre-incubated at 15°C for one week prior to the CUE measurement."

[Specific comment 15] *Line 482, what's the fixed effect and random effect in the linear mixed model?*

[Response]: We thank the reviewer for this reminder. We thank the reviewer for this question. In our linear mixed model, the fixed effects were the environmental and biotic factors (e.g., MAP, pH, C:N, microbial traits), while the random effect was sampling site, which accounts for the non-independence of the three replicates collected at each of the 40 locations along the transect. This has been clarified in the Methods section: "A linear mixed model was fitted with fixed effects of environmental and biotic factors and sampling site as the random effect along the transect."

Supplementary materials:

[Specific comment 16] *The text should be cited in the MS.*

[Response]: We thank the reviewer for this reminder. We have now ensured that all Supplementary Materials (Figures and Tables) are cited at their relevant points in the main text..

[Specific comment 17] *Fig. S6, in a priori model, the authors do not consider the effect of climate on plant, but the effect exists in the final model. Also, I think some effects are missed in a priori model such as soil properties to microbial traits and microbial traits to enzyme activity.*

[Response]: We are grateful to the reviewer for this exceptionally insightful comment on our conceptual model. We agree and have revised our a priori model (Fig. S6) accordingly. The following paths have been added to better align with established ecological theory and the reviewer's insight:

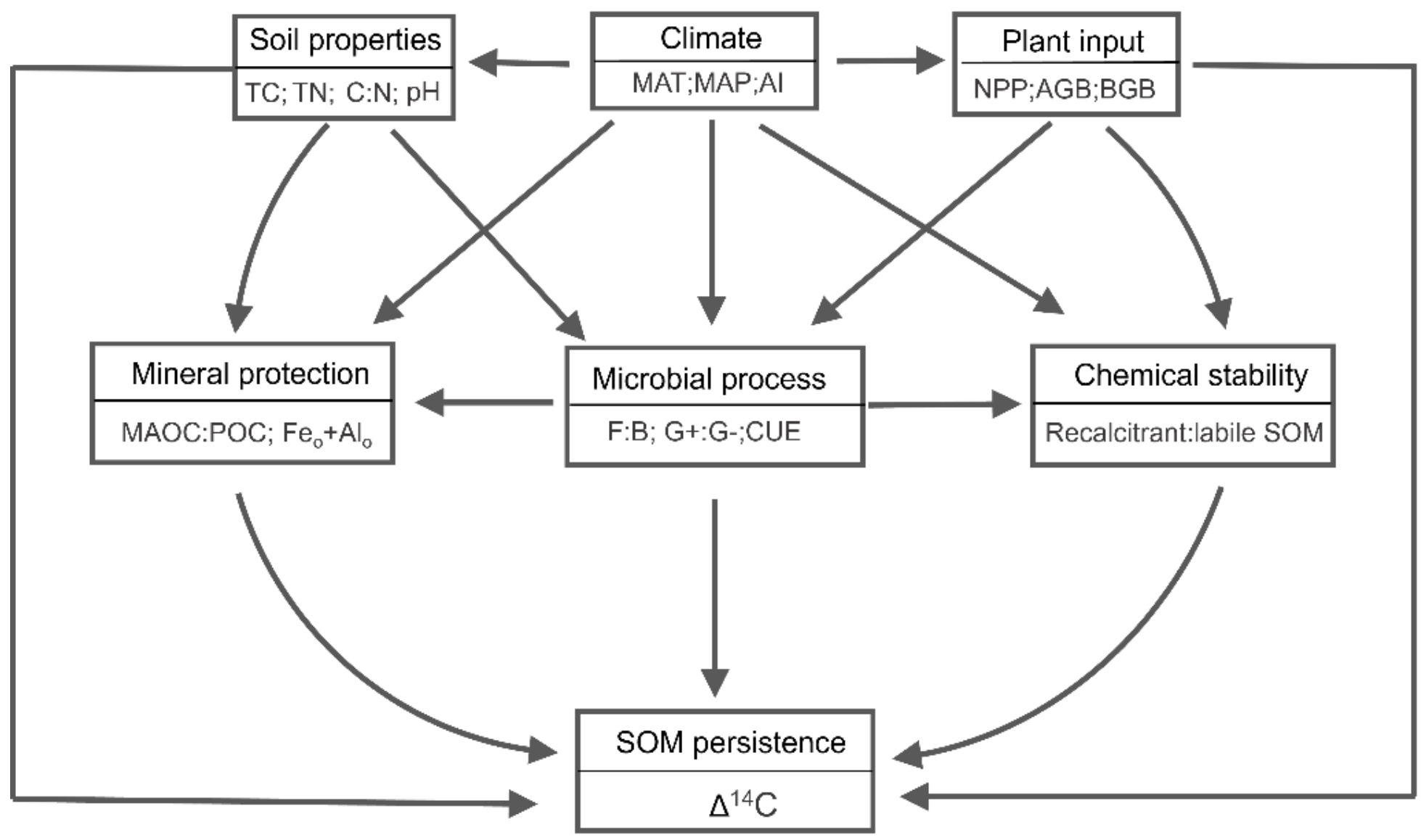

Once again, we extend our sincere gratitude to Reviewer #2 for their thorough and constructive feedback. We believe that by addressing all the comments, we have significantly improved the clarity, rigor, and impact of our manuscript. We hope that our revisions and responses are now found to be satisfactory.

Reviewer #1 (Remarks to the Author):

Comments to Authors

I have reviewed the revised manuscript entitled "Declines in Organic Matter Persistence with Increased Soil Carbon" by Zhao et al. As one of the original reviewers, I would like to commend the authors for their comprehensive and thoughtful responses to the previous comments. The manuscript has been significantly improved, and I am satisfied with the revisions.

Before final publication, I would like to suggest the following minor technical corrections to further enhance clarity and precision:

[Comment 1] *Line 90: Consider adding "by" before "enhanced," as the current phrasing appears grammatically incomplete. The sentence should be reviewed and revised for clarity.*

[Response]: Thank you for this helpful suggestion. During the revision of the Introduction, we substantially restructured this section and removed the original sentence containing the phrase "enhanced". As a result, the grammatical issue noted by the reviewer no longer applies in the revised manuscript. The current version has been carefully edited to ensure clarity and grammatical correctness throughout.

[Comment 2] *Line 143-144, The abbreviations Ald, Fed, Alo, and Feo are used here for the first time. To ensure readability for a broad audience, please provide their full names upon initial use.*

[Response]: We appreciate this suggestion. We have now provided the full names of these abbreviations upon their first appearance in the text. Specifically, Ald and Fed are defined as dithionite-extractable aluminum and iron oxides, respectively, while Alo and Feo are defined as oxalate-extractable aluminum and iron oxides.

We have revised the sentence at line 143-144 (now line 142-146) as follows: "Concurrently, we observed a significant shift in mineral protection capacity: the contents of dithionite-extractable Al and Fe oxides (Al_d and Fe_d) and Fe-bound organic carbon (OC–Fe) increased significantly with SOC (P < 0.05), whereas the molar ratios of oxalate-extractable

amorphous Al and Fe oxides (Al_o and Fe_o) to SOC, as well as (Al_d + Fe_d)/SOC, exhibited significant decreases (*P* < 0.01; Fig. 2a; fig. S4)."

[Comment 3] *Line 148-149, Although "recalcitrant" and "labile" are defined in the Method section, it would be beneficial to explicitly specify these terms in the main text as well. Please clearly indicate which compound classes (e.g., lignin, aliphatic biopolymers vs. sugars, proteins) are classified as recalcitrant and labile in the context of this study, supported by relevant references.*

[Response]: Thank you for this constructive comment. We have revised the Results section to explicitly specify the compound classes represented by recalcitrant and labile SOM. This clarification has been added directly in the main text to improve clarity and accessibility for readers.

The revised sentence was at line 149-153 as follows: "The ratio of recalcitrant (e.g., lignin-derived phenols, long-chain alkyl compounds, and polyaromatic hydrocarbons) to labile (e.g., polysaccharides and proteins) SOM significantly declined with increasing SOC along the transect (Fig. 1c; see Methods for details), indicating that the chemical recalcitrance of SOM decreased as SOC increased, which aligns with the observed rise in POM abundance"

Reviewer #2 (Remarks to the Author):

I acknowledge the authors' efforts and improvements during the last round of revision, but think they have not fully addressed some of the issues raised earlier.

[Response]: We thank the reviewer for their continued careful evaluation of our manuscript and for highlighting remaining points that required further clarification. We appreciate these constructive comments and have carefully revised the manuscript to address each concern in detail, as outlined below.

[Comment 1] *The authors state in the revised MS that their findings challenge the paradigm that C-rich soils are inherently more stable. I believe the traditional paradigm did not establish such relationship between carbon content and stability, and the authors' statement would be misleading to readers.*

[Response]: We thank the reviewer for this important clarification. We agree that the traditional soil carbon literature does not explicitly posit a universal paradigm that higher soil carbon content inherently confers greater SOM stability. Our original wording was therefore overly generalized and could be misleading.

In response, we have revised the relevant statements in the Introduction and Discussion to avoid framing our findings as challenging a broadly accepted paradigm. The revised text now emphasizes that SOC stocks reflect the balance between carbon inputs and losses, whereas long-term storage depends on SOM persistence regulated by plant, mineral, and microbial processes.

The revised sentence in the Introduction at line 51-53 was: "SOC stocks reflect the net balance between carbon inputs from plant production and carbon losses, primarily driven by microbial decomposition and respiration (Schmidt et al., 2011). While increasing plant carbon inputs can potentially enhance SOC stocks, the extent to which these inputs contribute to long-term storage is governed by the persistence of soil organic matter (SOM) (Schmidt et al., 2011; Cotrufo et al., 2013). Beyond carbon inputs, growing evidence suggests that SOM persistence depends less on chemical recalcitrance alone and more on the complementary effects of mineral

protection and the stabilization of microbial-derived compounds (Lehmann & Kleber, 2015a; Liang et al., 2017)"

We also have revised sentence in the Discussion at line 232-245 as following: "Our regional and global analyses consistently show that SOM persistence declines as SOC content increases, indicating that greater SOC content does not necessarily imply greater persistence…. This phenomenon suggests that the mechanisms driving SOC accumulation are not tightly coupled with those governing long-term persistence under high-input conditions."

[Comment 2] *Although the authors have added new analyses for $\Delta^{14}C$, how they use $\Delta^{14}C$ with MAOM and molecular-level recalcitrance to represent SOM persistence is still unclear. In the Introduction, the authors use a long paragraph to introduce MAOM, which gives impression that this manuscript mainly focuses on MAOM. However, current knowledge about $\Delta^{14}C$ is not included in the Introduction. In lines 124-138, the authors refer MAOM and molecular-level recalcitrance as potential mechanisms of SOM persistence, but in Fig. 2b, they use $\Delta^{14}C$ as predictor of physical and chemical composition. In addition, MAOM was not included in the SEM. These points are confused and the authors need to clearly organize these content and results.*

[Response]: We appreciate the reviewer's detailed comments on the conceptual organization of SOM persistence metrics. We acknowledge that, in the previous version, the roles of $\Delta^{14}C$, MAOM-related mineral protection, and molecular-level chemical composition were not clearly distinguished, which may have caused confusion. To address the reviewer's concerns, we have clarified the conceptual separation between indicators of SOM persistence and mechanistic controls on persistence across the Introduction, Results, and SEM framework.

(1) First, in the Introduction, we added a dedicated paragraph (second paragraph) explicitly introducing soil radiocarbon ($\Delta^{14}C$) as a time-integrated indicator of SOM persistence. At the same time, we reduced descriptive content on MAOM and microbial processes in the early part of the Introduction. The subsequent paragraph now focuses on mineral protection (including MAOM-related processes), molecular-

level recalcitrance, and microbial regulation as mechanistic pathways influencing persistence, rather than alternative persistence metrics.

We also added the following clarification at line 101-103: "In this study, Δ^{14}C is used as an integrative indicator of SOM persistence, while physical fractionation, mineral protection, molecular composition, and microbial traits are evaluated as mechanistic pathways regulating persistence."

(2) Second, Fig. 2b has been revised so that Δ^{14}C is no longer interpreted as a predictor of SOM physical or chemical properties. Instead, it is consistently treated as a response variable representing SOM persistence.

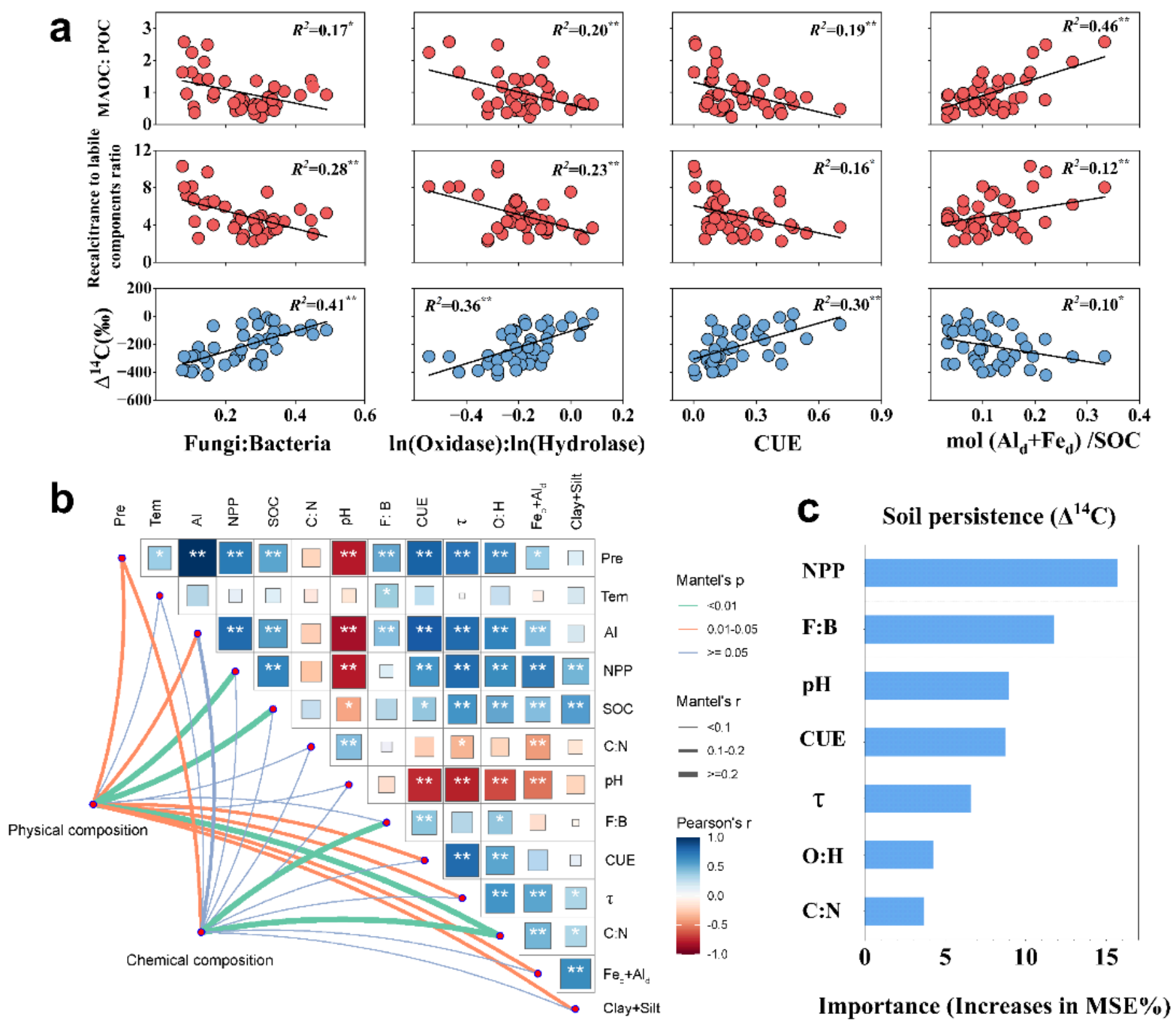

Fig. 2 Environmental variables influencing SOM persistence.

(3) Third, MAOM-related mineral protection is represented in the SEM by the MAOC:POC ratio, together with reactive Fe and Al oxides and Fe-associated organic carbon. Now, mineral protection is represented by MAOC:POC ratio, reactive Fe and Al oxides (Al_d + Fe_d; Fe_o + Al_o) and Fe-associated organic carbon (OC–Fe). Together, these variables reflect both the potential capacity for mineral–organic interactions and their realized contribution to SOM stabilization.

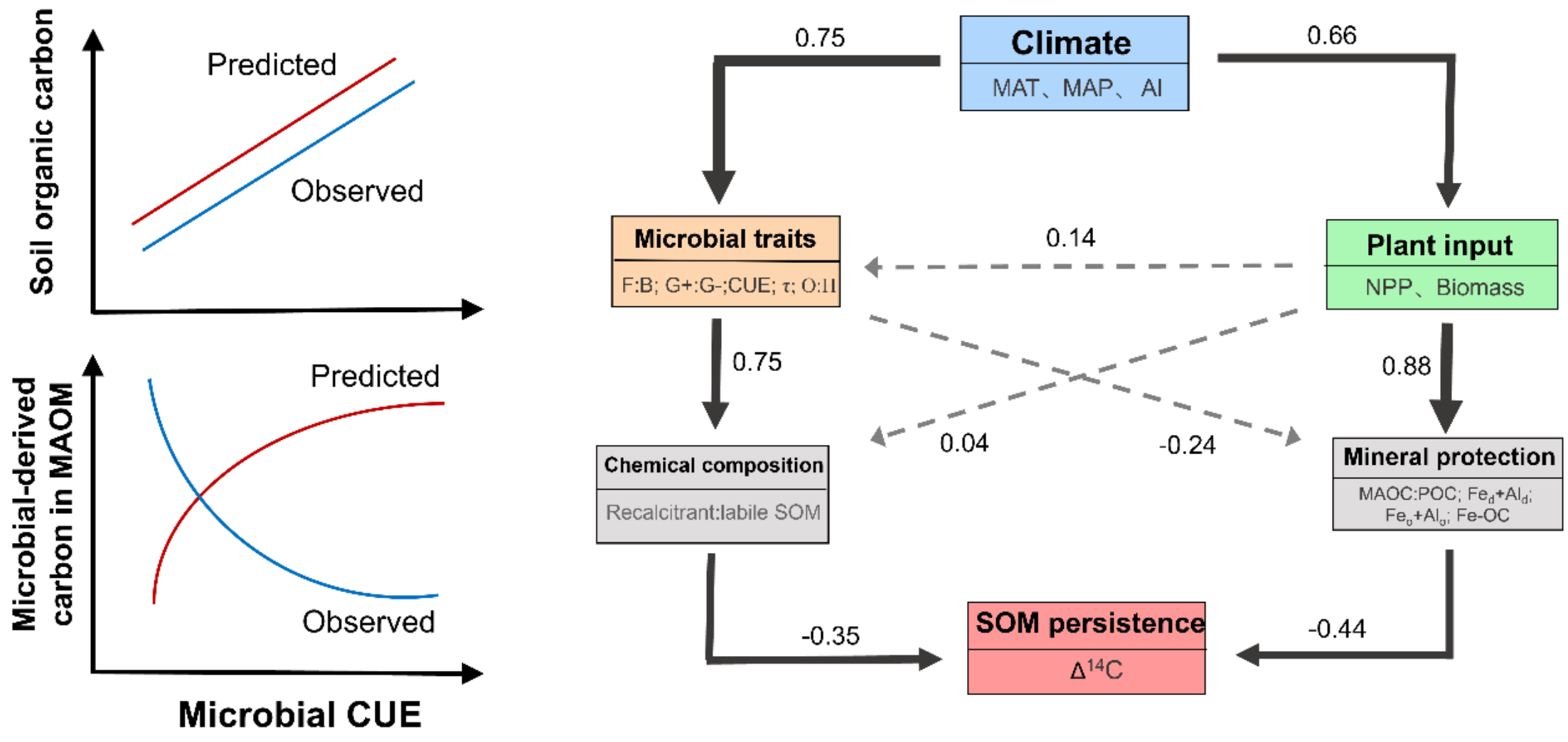

Fig. 2c Structural equation model revealing the influences of biotic and abiotic factors on SOM persistence

Together, these revisions align the conceptual framework, figures, and SEM structure, and clearly distinguish radiocarbon-based persistence indicators from the physical, chemical, and biological mechanisms that regulate SOM persistence.

[Comment 3] *Both random forest and SEM reveal the importance of NPP in affecting SOM persistence, but this effect is not discussed in current MS. Instead, the authors used long paragraphs to discuss microbial pathways driving MAOM recycling, which is not closely related to the main topic of the MS and lacks direct experimental evidence. Particularly, the statements regarding nutrient limitation need to be supported by direct results.*

[Response]: We appreciate the reviewer's insightful critique regarding the imbalance in the original Discussion. We agree that the importance of NPP, as consistently identified by both the Random Forest analysis and the SEM, was insufficiently emphasized, while the discussion of microbial MAOM recycling was overly extended and not always directly supported by empirical evidence. In the revised manuscript, we have substantially reorganized the Discussion to explicitly place plant carbon input (NPP) at the center of our conceptual framework for SOM persistence. The

main revisions are summarized as follows:

(1) We added a new paragraph (the third paragraph of the Discussion) that explicitly identifies NPP as an important predictor of SOM persistence. This paragraph explains that higher plant carbon inputs are associated with increased SOC accumulation but also with a greater proportion of fast-cycling carbon, as indicated by higher $\Delta^{14}C$ values and increased POM contributions. These patterns link NPP to the observed decline in radiocarbon-based SOM persistence ($\Delta^{14}C$).

(2) In response to the reviewer's concern, we have substantially shortened and refocused the discussion of microbial-driven MAOM recycling. Rather than treating microbial pathways as independent or speculative drivers, we now frame changes in microbial traits (e.g., enzyme allocation patterns, fungal-to-bacterial ratios, and microbial CUE) as biological responses to increased carbon supply driven by higher NPP. This revision ensures that microbial processes are explicitly linked to plant input effects revealed by the statistical analyses.

(3) Regarding nutrient limitation, we have removed speculative statements and now rely exclusively on patterns directly supported by our data. Specifically, we highlight the concurrent decline in leaf and soil C:N ratios alongside the increase in the NPP:TN ratio (fig. S11). This stoichiometric evidence indicates that ecosystem nitrogen demand increases more rapidly than soil nitrogen stocks under high-input conditions, consistent with intensified biological activity and faster SOM turnover.

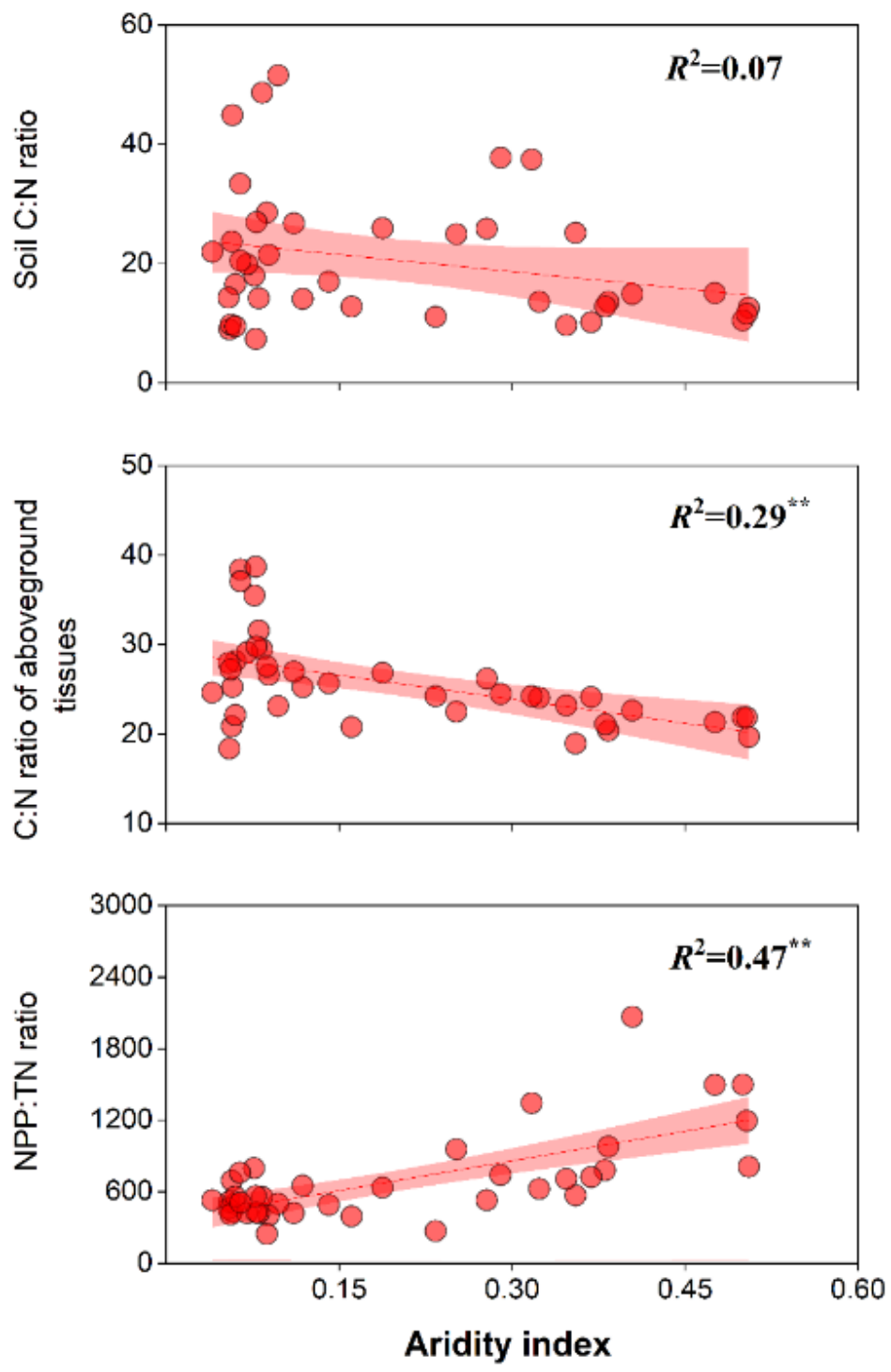

Supplementary Fig. 11 Stoichiometry of plant and soil along the transect.

We hope that these revisions can bring the Discussion into closer alignment with our empirical results and provide a more coherent, evidence-based explanation for the dominant role of NPP in regulating SOM persistence.

[Comment 4] *There are still errors in the revised MS. For example, line 217, it should be increase but not decrease. The arrow between chemical stability and SOM persistence was missed in a priori model in Fig. S9, but shown in the final SEM. These errors should be avoided in high quality papers in Nature Communications.*

[Response]: We sincerely apologize for these oversights and thank the reviewer for pointing them out. We have carefully corrected the identified errors, including revising “decrease” to “increase” in line 217 (now line 223) and ensuring consistency between the a priori model and the final SEM in Fig. S9.

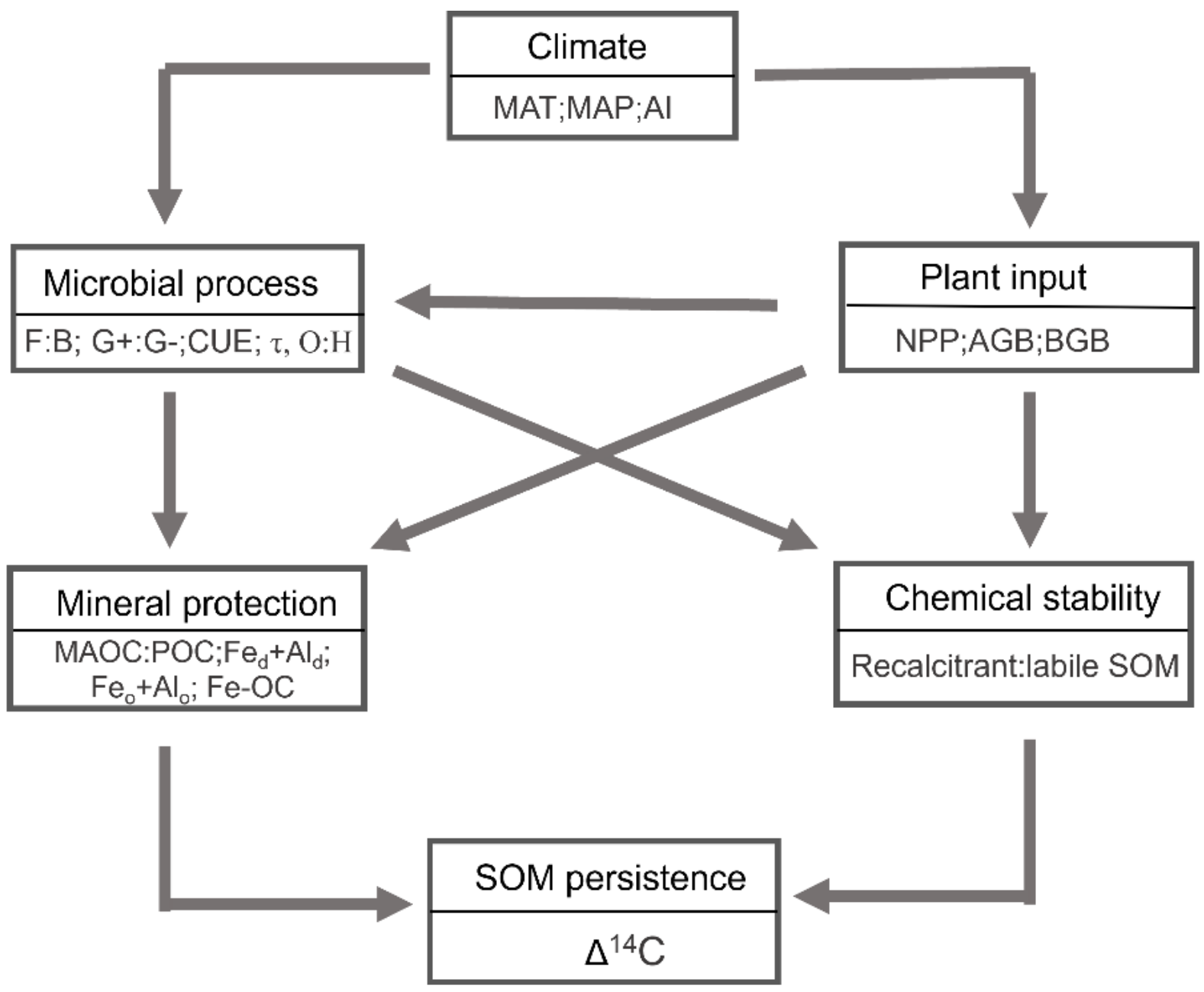

Supplementary Fig. 9 A priori model for piecewise structural equation modeling (SEM) analysis of soil organic matter persistence along the grassland transect.

In addition, we have conducted a thorough re-examination of the entire manuscript, figures, and supplementary materials to eliminate similar inconsistencies and ensure a high standard of accuracy and presentation appropriate.

Other revision:

In addition to the specific revisions made in response to the reviewers' comments, we have implemented several further improvements to enhance the overall clarity, coherence, and robustness of the manuscript.

(1) We revised the Abstract to more clearly articulate the central findings and revised conceptual framework of the study. In particular, the Abstract now explicitly emphasizes the radiocarbon-based decline in SOM persistence with increasing SOC across both the transect and global scales, highlights the decoupling between SOC content and persistence driven by the accumulation of young, fast-cycling carbon, and clarifies the key role of plant carbon inputs and microbial roles in regulating SOM persistence.

(2) We refined the hypothesis in the Introduction to ensure clearer alignment between the study objectives as following: "We hypothesized that SOM persistence would decline with increasing SOC content across soil carbon gradients, reflecting the increasing influence of plant carbon inputs and associated microbially mediated soil carbon turnover relative to mineral-associated stabilization."

(3) We conducted a careful revision to improve clarity, consistency, and precision in terminology, figure references, and cross-referencing between the main text and Supplementary Information. Minor language edits were also made for clarity and consistency.

Reviewer #1 (Remarks to the Author):

I have re-reviewed the revised manuscript by Zhao et al. The authors have responded thoughtfully to all prior comments, and the manuscript is greatly improved. I am happy to recommend it for publication.

Response: We sincerely thank the reviewer for the positive evaluation of our revised manuscript and for the constructive comments provided throughout the review process.

Reviewer #2 (Remarks to the Author): The authors have done a good job addressing the concerns raised in the previous round. I have the following minor comments on the revised manuscript.

Response: We sincerely thank the reviewer for the positive evaluation of our revised manuscript and for the helpful comments provided.

[Comment 1] *Line 120: Does this sentence imply that soil pH is higher in sites with higher NPP? Given that soil pH is typically negatively associated with SOC, this description is somewhat surprising.*

Response: We thank the reviewer for pointing out this error. We rechecked Table S1 and confirmed that the original description of soil pH was incorrect. Soil pH did not increase with the aridity index; instead, it showed the opposite trend along the transect.

We have revised the sentence at line 121-122 as: “Plant biomass, net primary productivity (NPP), soil total nitrogen (N), dissolved organic carbon, and available N (AN) increased with the aridity index, whereas soil pH showed the opposite trend (Table S1). “

[Comment 2] *Lines 137-138: I suggest mentioning here that microbial-derived carbon was determined for POM and MAOM fractions separately to make this information clearer.*

Response: We thank the reviewer for this helpful suggestion. We have revised the text to explicitly clarify that microbial-derived carbon was determined separately for the POM and MAOM fractions (see Methods),

thereby improving clarity.

The revised sentence at line 137-139 now reads as: "The content of microbial-derived carbon (estimated using amino sugars and determined separately in bulk soil as well as in the POM and MAOM fractions; see Methods) increased with SOC ($P < 0.05$; Fig. 3a)."

[Comment 3] *Lines 142-146: as mentioned by the authors, the contents of Ald, Fed and OC-Fe exhibited opposite patterns compared to the molar ratios. Which metrics are ultimately used to represent the capacity of mineral protection?*

Response: We thank the reviewer for this important comment. We agree that the contrasting trends between the absolute contents of Fe/Al oxides and their molar ratios relative to SOC may cause confusion. In our study, although the absolute contents of Al_d, Fe_d, and OC–Fe increased with SOC, these increases did not keep pace with the more rapid accumulation of SOC. Therefore, the molar ratios of reactive oxides to SOC (e.g., ($Al_d + Fe_d$)/SOC and ($Al_o + Fe_o$)/SOC) better reflect the relative capacity of the mineral matrix to stabilize organic carbon.

We have revised the sentence at line 144-150 as: "Concurrently, we observed contrasting patterns in mineral-related indicators: although the contents of dithionite-extractable Al and Fe oxides (Al_d and Fe_d) and Fe-bound organic carbon (OC–Fe) increased significantly with SOC ($P < 0.05$), the molar ratios of reactive Fe/Al oxides to SOC (e.g., ($Al_d + Fe_d$)/SOC and (Al_o + Fe_o)/SOC) decreased significantly ($P < 0.01$; Fig. 2a; Fig. S4). This indicates that, despite increasing absolute oxide contents, the relative capacity of the mineral matrix to stabilize SOC declined as SOC increased. Accordingly, oxide-to-SOC ratios were used as indicators of mineral protection capacity in subsequent analyses. "

[Comment 4] *Lines 173-176: the sentences are incomplete*

Response: We thank the reviewer for pointing this out. We found that the original sentence contained an incomplete parallel structure. We have revised it to clearly describe the negative correlations of MAOC:POC with F:B, O:H,

and microbial CUE, and its positive correlation with the molar ratio of (Ald + Fed) to SOC.

Now the sentence reads at line 178-181 as: "In particular, SOM physical stability (MAOC:POC) was negatively correlated with the fungal-to-bacterial ratio (F:B; R^2 = 0.17, P < 0.05), the oxidase-to-hydrolase ratio (O:H; R^2 = 0.20, P < 0.05), and microbial CUE (R^2 = 0.19, P < 0.05), but positively correlated with the molar ratio of (Al_d + Fe_d) to SOC (R^2 = 0.46, P < 0.05)."

Other revisions:

In addition, we carefully reviewed the entire manuscript and made further revisions to improve clarity, consistency, and language throughout.

Lines 140–143: The sentence "Furthermore, the ratio of microbial-derived carbon stored in the MAOM fraction compared to the POM fraction also decreased as SOC increased (P < 0.05; Fig. 3a; Fig. S3). Collectively, these results demonstrate that the higher SOC is primarily associated with increased POM, alongside a declining proportion of mineral-stabilized microbial-derived carbon" has been revised as:

"Furthermore, the ratio of microbial-derived carbon in MAOM to that in POM also decreased as SOC increased (P < 0.05; Fig. 3a; Fig. S3). Collectively, these results indicate that higher-SOC soils are characterized by a greater proportion of POM and a lower relative contribution of mineral-stabilized microbial-derived carbon."

Lines 166–168: The sentence "Further analysis of CUE (see Fig. S8 and explanation) indicated that higher CUE reflected enhanced microbial growth (R2 = 0.67) rather than decreased microbial respiration (R^2 = 0.11)" has been revised as:

"Further analysis of CUE (see Fig. S8 and explanation) indicated that higher CUE reflected enhanced microbial growth (R^2 = 0.67) more strongly than reduced microbial respiration (R^2 = 0.11)."

Lines 189–190: The sentence "We observed a significant positive correlation

between microbial CUE and SOC, as well as microbial-derived carbon, across the transect” has been revised as:

“We observed a significant positive correlation between microbial CUE and both SOC and microbial-derived carbon across the transect.”

Lines 324–326: The sentence “Spanning a length of 1500 km, the transect exhibits varying mean annual precipitation (ranging from 50 to 650 mm) and mean annual temperature (ranging from −3.6 to 2.3 °C) levels from east to west (http://www.worldclim.org).” has been revised as:

“Spanning 1,500 km, the transect exhibits varying mean annual precipitation (ranging from 50 to 650 mm) and mean annual temperature (ranging from −3.6 to 2.3 °C) from west to east (http://www.worldclim.org).”

Lines 350–352: The sentence “Soil inorganic carbon was measured after removing organic carbon by heating separate sample aliquots at 450°C for 16 hours, as well as density fractionation (see below).” has been revised as:

“Soil inorganic carbon was measured in both bulk soil and fractionated samples (see below) after removing organic carbon from separate sample aliquots by heating at 450 °C for 16 h.”
